# Social frailty and the risk of progression from health to physical, psychological, and cognitive multimorbidity: a prospective multi-cohort study

**DOI:** 10.1186/s12916-026-04761-8

**Published:** 2026-03-06

**Authors:** Yaguan Zhou, Htet Lin Htun, Mika Kivimäki, Zhengluan Liao, Xiaolin Xu, Yi Guo

**Affiliations:** 1 School of Public Health, The Second Affiliated Hospital, Zhejiang University School of Medicine, Hangzhou, Zhejiang China; 2 The Key Laboratory of Intelligent Preventive Medicine of Zhejiang Province, Hangzhou, Zhejiang China; 3 School of Public Health and Preventive Medicine, Monash University, Melbourne, Australia; 4 Brain Sciences, University College London, London, UK; 5 Clinicum, University of Helsinki, Helsinki, Finland; 6 Department of Psychiatry, Zhejiang Provincial People’s Hospital, Hangzhou, Zhejiang China; 7 School of Public Health, Faculty of Medicine, The University of Queensland, Brisbane, Australia; 8 Department of General Practice and International Medicine, The Second Affiliated Hospital of Zhejiang University School of Medicine, Hangzhou, Zhejiang China; 9 Department of Neurology, Epilepsy Center, The Second Affiliated Hospital of Zhejiang University School of Medicine, Hangzhou, Zhejiang China

**Keywords:** Social frailty, Physical, psychological, and cognitive conditions, Multimorbidity, Multi-cohort

## Abstract

**Background:**

Social frailty, a state of vulnerability to social risk factors, has been linked to adverse physical, psychological, and cognitive outcomes. However, evidence on its role in the progression of multimorbidity across these domains is limited. This study examined social frailty in relation to transitions from a healthy state to physical, psychological, and cognitive conditions and to multimorbidity.

**Methods:**

In this multi-cohort study across 24 countries in the USA, Europe, and Asia, middle-aged and older individuals with no preexisting physical, psychological, or cognitive conditions were included. Social frailty, assessed through baseline questionnaires, was defined across four domains: general resources, social behaviors, social resources, and basic social needs. Physical, psychological, and cognitive conditions were ascertained at baseline and reassessed four times during a mean follow-up of 7 years. Multi-state models and the group-based multi-trajectory model were used to examine the role of social frailty in the development of physical, psychological, and cognitive conditions and their progression to multimorbidity.

**Results:**

Among the 7119 participants (mean age, 59.6 years [SD 8.5]; 3575 [50.2%] women), 778 (10.9%) were socially frail at baseline. During 49,648 person-years at risk, 2981 (41.9%) individuals progressed from a healthy state to one physical, psychological, or cognitive condition, and 1592 (22.4%) progressed to multimorbidity. Compared with socially robust participants, those with social frailty had a higher risk of progressing to any of these conditions (hazard ratio, 1.30; 95% CI, 1.17–1.44) and to multimorbidity (2.10, 1.67–2.65). Four outcome trajectories were identified, including “stably no physical, psychological or cognitive conditions” (48.0%), “increased physical conditions” (16.4%), “increased physical and psychological conditions” (8.7%), and “increased physical and cognitive conditions” (26.9%). Social frailty was associated with more than a twofold increase in risk for the latter three trajectories (odds ratios 2.34, 1.76–3.09, 2.27, 1.63–3.15 and 2.69, 2.11–3.44, respectively). The strongest associations were observed for frailty in the domains of general resources and basic social needs.

**Conclusions:**

Social frailty is associated with faster transitions from a healthy state to physical, psychological, and cognitive conditions and further progression to multimorbidity. Early identification and interventions to address social frailty may help improve health in middle-aged and older adults.

**Supplementary Information:**

The online version contains supplementary material available at 10.1186/s12916-026-04761-8.

## Background

Frailty is a complex state that represents a preliminary stage of increased vulnerability and need for long-term care [1]. Although previous research mainly focused on the physical aspects of frailty, recent studies have increasingly emphasized its social aspects [2]. According to the Social Production Function (SPF) theory, people produce their own well-being by trying to optimize achievement of universal goals, within the set or resources and constraints they face [3]. The SPF theory specified social needs of people for affection, behavioral confirmation, and status [4]. When the resources are limited or insufficient, people may fail to fulfill these social needs and become socially frail. Based on the SPF theory, a scoping review summarized evidence from previously published studies and constructed an integrated conceptualization of social frailty, including four distinct domains: general resources, social behaviors, social resources, and basic social needs [4]. General resources refer to resources for fulfilling social needs (e.g., educational level, income, and wealth). Social behaviors refer to activities that are likely to be exploited for social need fulfillment (e.g., volunteering, occupation, and social participation). Social resources refer to resources with interaction with others to fulfill social needs (e.g., marital status, family ties, and social network size). Notably, social resources point to the structural and objective foundation by which people are able to interact with others, while social behaviors emphasize on the subjective initiatives and quality of people to interact with others. These three domains are the basis to fulfill basic social needs, the fourth domain, referring to the needs for affection, behavioral confirmation, and status according to the SPF theory.

The prevalence of social frailty across various settings and subpopulation is estimated to range from 18.8% to 47.3% among adults aged ≥ 60 years, which varies significantly using different definitions [5]. Cross-sectional studies have linked social frailty (defined by five categories: inability to help others, limited social participation, loneliness, financial difficulty, and living alone) to a higher prevalence of many chronic physical conditions, including cardiovascular and cerebrovascular diseases [6], as well as to multimorbidity [7]. A systematic review of longitudinal studies further suggested that social frailty is associated with a higher risk of functional disability, poor mental health, and reduced neuropsychological function [8]. In a 4-year follow-up study of 3538 older Japanese adults, social frailty was associated with depressive symptoms [9], and similar findings have been reported in a prospective study from China [10]. Other studies have shown that older adults with social frailty experience faster cognitive decline [11] and a higher risk of incident dementia [12]. These abovementioned studies have contained different domains of social frailty, including general resources (e.g., financial difficulty), social behaviors (e.g., not visiting friends), social resources (e.g., living alone), and basic social needs (e.g., family disharmony, having no one to talk to and feeling lonely), providing convincing evidence on the relationship between social frailty and many adverse health outcomes.

Emerging evidence has shown that physical, psychological, and cognitive conditions may create a vicious cycle where each condition exacerbates the others. Reciprocal relationships between physical conditions and psychological illnesses, such as depression and anxiety, have been well documented [13–15]. Evidence from Latin America shows that chronic physical conditions contribute to poorer late-life cognition [16], while European data confirm an association between physical conditions and dementia risk, particularly in middle-aged individuals [17]. Multimorbidity involving physical, psychological, or cognitive conditions affects an estimated 8.1%–33.9% of adults aged 45 years or older worldwide [18]. Longitudinal data from 24 countries and 20,250 adults with at least one chronic physical condition indicate that 39.2% progress to psychological and cognitive multimorbidity, the highest incidence in those with socioeconomic disadvantage [19]. However, the role of social frailty in the development of multimorbidity involving these conditions remains largely unknown. Studies from Asian populations have reported associations of social frailty with physical health, depressive symptoms, and cognitive dysfunction [20, 21], but without accounting for the temporal sequence of these outcomes. On this basis, several research has revealed the role of social frailty in the progression of multiple conditions, even when individuals have occurred one of them. For example, analysis of 576 survivors of intercerebral hemorrhage observed that social vulnerability was independently associated with depression [22]. In addition, a Chinese longitudinal analysis of 5555 older adults investigated the moderating effects of psychological resilience on the relationship between social frailty and cognitive outcomes. This study found that compared to individuals with social robustness and high psychological resilience, those with social frailty and low psychological resilience demonstrated higher odds ratios of developing cognitive impairment and greater cognitive decline [23]. Given the complex interplay among chronic physical, psychological, and cognitive conditions in older adults, a systematic approach to their transition is needed to better understand the implications of social frailty. However, there is scanty evidence on how social frailty influence the progression to these conditions and their multimorbidity in a longitudinal study design. Furthermore, previous studies have been conducted in a single country or a limited region with different study periods and analytical strategies, which precluded comparing results and limited the generalizability of these findings.

To address these limitations, we used harmonized data from four cohort studies across 18 countries and systematically examined how social frailty relates to transitions from a healthy state to physical, psychological, and cognitive conditions and subsequently to multimorbidity. We sought to identify the predicting ability of social frailty for the progression to complex multimorbidity, which are applicable to various populations, and further to inform the better implementation of long-term care and primary and secondary prevention strategies.

## Methods

### Study design and participants

This multi-cohort study adhered to the Strengthening the Reporting of Observational Studies in Epidemiology (STROBE) reporting guideline. Individual-level data were harmonized from four sister cohort studies from the Global Aging, Health and Policy program: the US Health and Retirement Study (HRS), the English Longitudinal Study on Ageing (ELSA), the Survey of Health, Ageing and Retirement in Europe (SHARE), and the China Health and Retirement Longitudinal Study (CHARLS). These studies recruited a nationally representative sample of middle-aged and older adults, sharing similar protocols, and conducted surveys every 2 or 3 years. Additional details for each study have been provided elsewhere [24–27]. To allow cross-study comparisons, we used data in a consistent period in each study, including waves 11 to 15 of HRS (2012–2021), waves 5 to 9 of ELSA (2010–2019), waves 5 to 9 of SHARE (2012–2021), and waves 1 to 5 of CHARLS (2011–2021) (Additional file 1: Table S1).

**Fig. 1:**
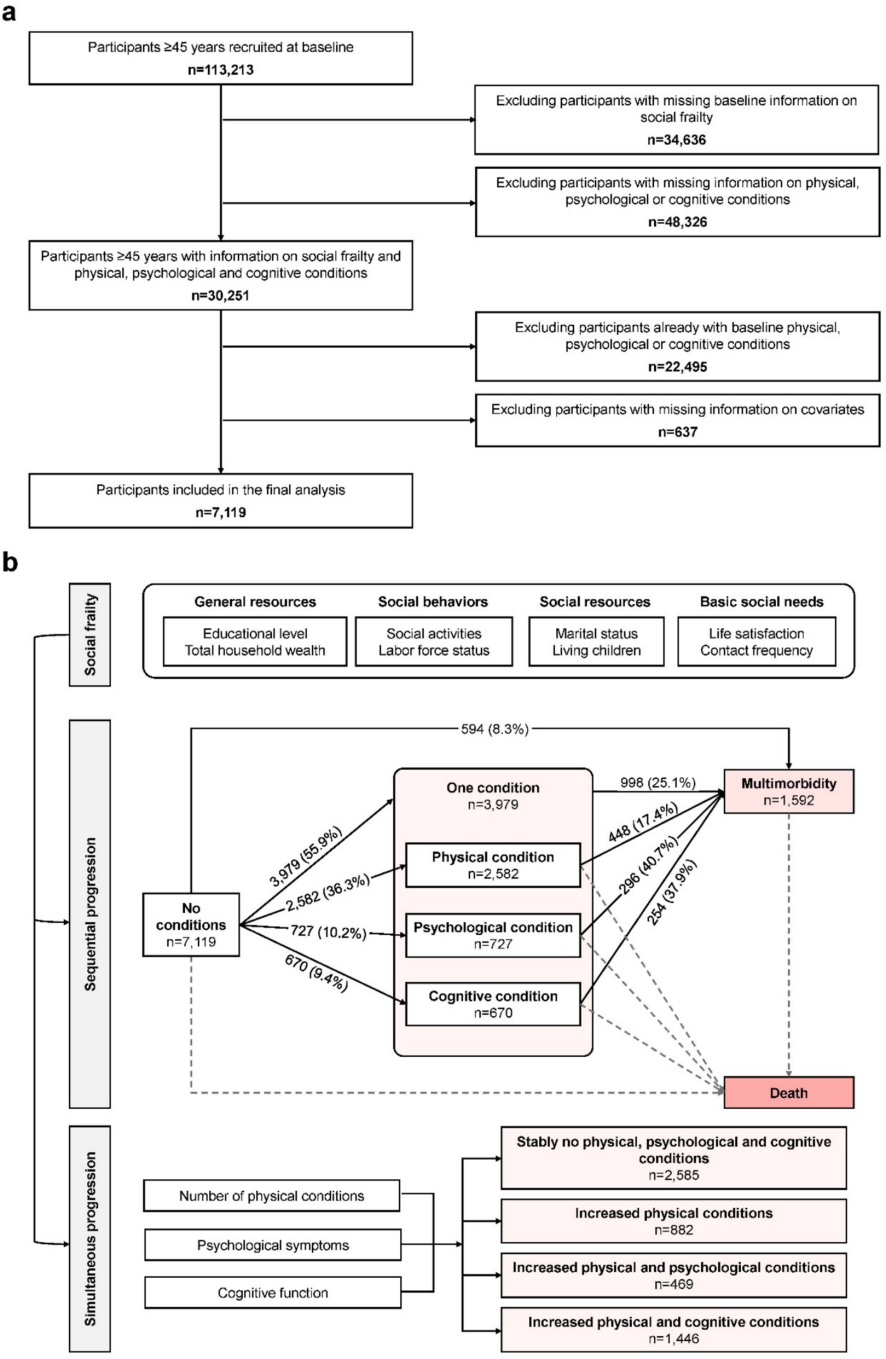
Flowchart of study design. **a** Participant’s selection. **b** The analytic approach

Participants were included if they (1) provided information on social frailty and physical, psychological, and cognitive conditions; (2) had no preexisting physical, psychological, or cognitive conditions at baseline; and (3) provided information on covariates (Fig. 1a). All participating studies were approved by Institutional Review Boards, and the respondents provided written informed consent.

### Social frailty

We assessed social frailty at baseline, defined as wave 11 in the HRS, wave 5 in the ELSA and SHARE, and wave 1 in the CHARLS. According to a previous scoping review, social frailty was assessed through domains of general resources, social behaviors, social resources, and basic social needs [4]. Considering the data availability, we constructed each domain of social frailty by two variables.

1. General resources: Educational level and total household wealth were dichotomized to measure the frailty in general resources. Participants lower than the first tertile of educational level or total household wealth were considered frail in general resources (scored 1); otherwise, they were non-frail (scored 0).
2. Social behaviors: Frequency of social activities and labor force status were used to construct the level of social behaviors. Participants reporting no monthly social activities or unemployment were considered frail in social behaviors (scored 1); otherwise, they were non-frail (scored 0).
3. Social resources: Marital status and living children were used to construct the level of social resources. Participants reporting not married/partnered or no living children were considered frail in social resources (scored 1); otherwise, they were non-frail (scored 0).
4. Basic social needs: Information on participants’ satisfaction with life and frequency of contact with children was captured to construct the level of basic social needs. Participants reporting lower than the mean score of satisfaction with life or no weekly contact with children were considered frail in basic social needs (scored 1); otherwise, they were non-frail (scored 0). Detailed descriptions of the assessment of social frailty in each study are provided in Additional file 1: Table S2.

Social frailty was categorized based on the summed score of these domains: socially robust (first tertile, score 0 or 1), social pre-frailty (second tertile, score 2), and social frailty (third tertile, score 3 or 4) [28]. Social pre-frailty, a status that lies between social frailty and robustness, has been widely observed to be associated with adverse health outcomes [23, 29, 30].

### Physical, psychological, and cognitive conditions and multimorbidity

Physical, psychological, and cognitive conditions were assessed at baseline and at four follow-up waves (waves 11–15 in HRS, waves 5–9 in ELSA and SHARE, and waves 1–5 in CHARLS). Participants with any of these conditions at baseline were excluded from the analysis.

Physical conditions were defined as self-reported diagnoses of at least one of seven age-related diseases: hypertension, diabetes, cancer, chronic lung disease, heart disease, stroke, and arthritis.

The presence of the psychological condition was ascertained using study-specific assessments: the 8-item version of the Center of Epidemiological Studies Depression Scale (CESD-8) in HRS and ELSA, the 12-item version of the European-Depression Scale (EUROD-12) in SHARE, and the 10-item version of the Center of Epidemiological Studies Depression Scale (CESD-10) in CHARLS. All instruments have been validated in the respective older populations. Study-specific and validated cut-off values were applied to identify participants with psychological conditions. To assess symptom severity, scores were standardized into z-scores by subtracting the baseline sample mean and dividing by standard deviation, with a higher z-score indicating more severe psychological symptoms.

In each study, the cognitive function of participants was assessed through the following subtests: episodic memory (immediate and delayed word recall), orientation (recall test of time, place and person), executive function (serial 7’s subtraction test in HRS, SHARE, and CHARLS), and semantic fluency (animal fluency test in ELSA). The score of each subtest was converted into the z-score through subtraction by the average value of the study sample at baseline and then division by the standard deviation. Participants with a z-score lower than -1.5 in at least one cognitive subtest were regarded as having the cognitive condition [19]. To investigate the level of cognition function, we calculated the composite cognitive score as the mean z-score of all subtests, with a higher score indicating better cognitive function.

Detailed descriptions of the assessment of physical, psychological, and cognitive conditions in each study are provided in Additional file 1: Table S2. Physical, psychological, and cognitive multimorbidity were defined based on the occurrence of physical, psychological, and/or cognitive conditions and included four categories (physical-psychological multimorbidity, physical-cognitive multimorbidity, psychological-cognitive multimorbidity, and physical-psychological-cognitive multimorbidity), in accordance with previous studies from our research team [18, 19].

### Covariates

Covariates included demographic characteristics (age, sex, and study), lifestyle factors (current smoking and alcohol consumption), and body mass index (BMI), which were assessed at the baseline survey. Smoking status was dichotomized by asking participants whether they smoked at the present time, with answers of “yes” (current smoking) or “no” (never smoking or quitting smoking). Self-reported alcohol consumption on ≥ 1 days per week was regarded as weekly or more frequent drinking, otherwise less than weekly drinking. BMI was derived by dividing weight (kg) by the square of height (m^2^), which was categorized into four groups: underweight (< 18.5 kg/m^2^), normal (18.5–24.9 kg/m^2^), overweight (25.0–29.9 kg/m^2^), and obese (≥ 30.0 kg/m^2^).

### Statistical analysis

The baseline characteristics of participants were described as number (percentage) according to social frailty at baseline and the progression to physical, psychological, and cognitive conditions and multimorbidity during follow-up. Chi-squared tests were used to compare the between-group differences.

We conducted multi-state models to examine the association between social frailty and the progression to physical, psychological, and cognitive conditions and multimorbidity. The multi-state model is an extension of the competing risk model, which can also explore how the exposures of interest influence transitions to intermediate events in survival data [31]. In the present analysis, death was censored as a competing event and not modelled as a direct outcome. For those reporting two or three of physical, psychological, and cognitive conditions at the same wave, sequences of these conditions could not be established, and a direct transition from baseline to multimorbidity was additionally set. Therefore, the transition pattern of physical, psychological, and cognitive conditions and multimorbidity involved three temporal paths: (1) from no physical, psychological, or cognitive conditions to one of the conditions; (2) from no physical, psychological, or cognitive conditions to multimorbidity; and (3) from one of the conditions to multimorbidity (Fig. 1b). Incidence rates of the abovementioned transitions by social frailty were calculated as the number of new events divided by the time at risk (1000 person-years), with 95% confidence intervals (CIs) estimated according to the Poisson distribution. Hazard ratios (HRs) and 95% CIs for all these transitions were estimated by social frailty adjusted for age, sex, study, current smoking, alcohol consumption, and BMI. The HRs and 95% CIs for the association of each domain of social frailty and each measurement of social frailty domains with the transitions between no physical, psychological, or cognitive conditions and multimorbidity were also evaluated, adjusted for the same sets of covariates. In the additional analysis, we specified the first occurring conditions in multi-state models and generated seven temporal paths: (1) from baseline to the physical condition, (2) from baseline to the psychological condition, (3) from baseline to the cognitive condition, (4) from baseline to multimorbidity, (5) from the physical condition to multimorbidity, (6) from the psychological condition to multimorbidity, and (7) from the cognitive condition to multimorbidity. Subgroup analyses by age groups, sex, and study were performed, and random-effects meta-analyses were conducted to summarize study-specific HRs and 95% CIs for each transition, with the between-study heterogeneity tested using *I*^2^ statistics.

The group-based multi-trajectory model (GBMTM) was further performed to visualize the simultaneous changes of number of physical conditions, psychological symptoms, and cognitive function (Fig. 1b). Participants with outcome information at three or more time points were included in the GBMTM. This method allows us to identify groups of participants who share similar trajectories of indicators over time. In order to determine the trajectory groups that best fit the data, we used the PROC TRAJ procedure for SAS, yielding a probability for each subject of being in each trajectory group. The optimal number and fitted shape of trajectories were identified based on Akaike information criterion (AIC) and Bayesian information criteria (BIC) [32]. Additionally, we assessed the average posterior probability (above 0.7) and the predicted probability of group membership (above 5%) for further validation [33]. Individuals were assigned to the group having the highest posterior probability. Multinomial logistic regression models were performed to examine the association between social frailty and trajectories of physical, psychological, and cognitive conditions. To minimize the bias caused by missing data, we applied multiple imputations via chained equations to impute missing values in outcomes, with the missingness assumed at random and imputed five times. Odds ratios (ORs) and 95% CIs were reported after adjusting covariates including age, sex, study, current smoking, alcohol consumption, and BMI. Subgroup analyses by age groups, sex, and study were also conducted, and random-effects meta-analyses were applied to summarize study-specific ORs and 95% CIs for each trajectory group, with the between-study heterogeneity tested using *I*^2^ statistics.

All tests in this analysis were two-sided with a significance level of *p* < 0.05. Statistical analyses were conducted using SAS (Version 9.4) and RStudio (Version 4.4.1).

## Results

A total of 113,213 participants aged ≥ 45 years were recruited at baseline. We excluded individuals with missing information on exposures (*n* = 34,636) and outcomes (*n* = 48,326), with preexisting physical, psychological, or cognitive conditions at baseline (*n* = 22,495) and with missing information on covariates (*n* = 637), yielding 7119 participants in the final analysis (Fig. 1a).

There were 171 (2.4%) participants from the USA, 1500 (21.1%) participants from China, and 1272 (17.9%) from the UK, and 4176 (58.7%) from 15 other European countries (Additional file 1: Table S1).

The baseline characteristics of the participants according to social frailty at baseline are summarized in Table 1. The mean [± standard deviation (SD)] age of the 7119 participants was 59.6 (± 8.5) years, and 3575 (50.2%) were female. There were 778 (10.9%) participants being socially frail and 4937 (69.3%) socially robust. Participants with social frailty were more likely to be older and female and more likely to report current smoking, less drinking, and a BMI of < 18.5 or ≥ 30 kg/m^2^.

**Table 1:** Baseline characteristics of included participants according to social frailty (*n* = 7119)

	Total (*n* = 7119)	Social frailty			*p*-value
		No social frailty (*n* = 4937)	Social pre-frailty (*n* = 1404)	Social frailty (*n* = 778)	
**Age group (years)**					< 0.001
45–54	2228 (31.3)	1650 (74.1)	381 (17.1)	197 (8.8)	
55–64	2968 (41.7)	2050 (69.1)	594 (20.0)	324 (10.9)	
65–74	1519 (21.3)	1018 (67.0)	329 (21.7)	172 (11.3)	
75	404 (5.7)	219 (54.2)	100 (24.8)	85 (21.0)	
**Sex**					0.001
Male	3544 (49.8)	2504 (70.7)	701 (19.8)	339 (9.6)	
Female	3575 (50.2)	2433 (68.1)	703 (19.7)	439 (12.3)	
**Current smoking**					< 0.001
No	5646 (79.3)	4034 (71.4)	1059 (18.8)	553 (9.8)	
Yes	1473 (20.7)	903 (61.3)	345 (23.4)	225 (15.3)	
**Alcohol consumption**					< 0.001
Less than weekly drinking	3380 (47.5)	2152 (63.7)	765 (22.6)	463 (13.7)	
Weekly drinking or more	3739 (52.5)	2785 (74.5)	639 (17.1)	315 (8.4)	
**Body mass index (kg/m**^**2**^**)**					0.028
< 18.5	117 (1.6)	75 (64.1)	27 (23.1)	15 (12.8)	
18.5–23.9	3495 (49.1)	2480 (71.0)	664 (19.0)	351 (10.0)	
24.0–29.9	2738 (38.5)	1879 (68.6)	551 (20.1)	308 (11.2)	
30.0	769 (10.8)	503 (65.4)	162 (21.1)	104 (13.5)	

Social frailty is measured by four domains: general resources (scored 0–1), social behaviors (scored 0–1), social resources (scored 0–1), and basic social needs (scored 0–1); higher score of each domain means higher degree of frailty. Summed scores of 2 and > = 3 from four domains (general resources, social behaviors, social resources, and basic social needs) are then set as social pre-frailty and social frailty, respectively

### Transitions from a healthy state to physical, psychological, and cognitive conditions and multimorbidity

During follow-up of 49,648 person-years (mean follow-up 6.97 years), a total of 3979 (55.9%) individuals transitioned from no physical, psychological, or cognitive conditions to one of these conditions, and 998 (25.1%) further transitioned to multimorbidity. In addition, 8.3% of the participants (*n* = 594) directly transitioned from no physical, psychological, or cognitive conditions to multimorbidity (Fig. 1b, Additional file 1: Table S3).

Individuals with social pre-frailty and social frailty had higher incidence rates of all transitions between no physical, psychological, or cognitive conditions and multimorbidity than those with no social frailty (Table 2). For example, the incidence rates of transition from no physical, psychological, or cognitive conditions to multimorbidity were 10.08 (95% CI = 9.08 to 11.20), 14.41 (95% CI = 12.15 to 17.07), and 20.78 (95% CI = 17.06 to 25.28) per 1000 person-years in participants with no social frailty, social pre-frailty, and social frailty, respectively. Compared to no social frailty, both social pre-frailty and social frailty were associated with transitions from no physical, psychological, or cognitive conditions to one of these conditions (social pre-frailty: HR = 1.24, 95% CI = 1.14 to 1.34; social frailty: HR = 1.30, 95% CI = 1.17 to 1.44) and directly to multimorbidity (social pre-frailty: HR = 1.45, 95% CI = 1.19 to 1.78; social frailty: HR = 2.10, 95% CI = 1.67 to 2.65), independently of covariates. With each additional score of social frailty, the corresponding hazard ratios of transitions to one condition and directly to multimorbidity rose by 11% (HR = 1.11, 95% CI = 1.08 to 1.15) and 33% (HR = 1.33, 95% CI = 1.24 to 1.43). For the transition from one of physical, psychological, and cognitive conditions to multimorbidity, associations with social pre-frailty (HR = 1.23, 95% CI = 1.06 to 1.44) and social frailty (HR = 1.23, 95% CI = 1.00 to 1.51) were also observed. In addition, a dose–response relationship of score of social frailty existed, with HR of 1.10 (95% CI = 1.04 to 1.17) per 1-point increase in the score.

**Table 2:** Incidence rates and hazard ratios for the association between social frailty and progressions of physical, psychological, and cognitive multimorbidity (*n* = 7119, 49,648 person-years)

	Incidence rate per 1000 person-years (95% CI)	HR (95% CI)
***No physical, psychological, or cognitive conditions to one of the conditions***		
**Social frailty**		
No	76.57 (73.83, 79.40)	Ref
Social pre-frailty	88.04 (82.44, 93.98)	1.24 (1.14, 1.34)
Social frailty	90.67 (82.85, 99.13)	1.30 (1.17, 1.44)
Per score increase	/	1.11 (1.08, 1.15)
**Domains of social frailty**		
General resources	87.51 (82.76, 92.50)	1.19 (1.10, 1.28)
Social behaviors	85.04 (81.30, 88.94)	1.11 (1.04, 1.19)
Social resources	84.09 (77.30, 91.41)	1.01 (0.92, 1.11)
Basic social needs	86.99 (81.76, 92.53)	1.17 (1.08, 1.26)
***No physical, psychological, or cognitive conditions to multimorbidity***		
**Social frailty**		
No	10.08 (9.08, 11.20)	Ref
Social pre-frailty	14.41 (12.15, 17.07)	1.45 (1.19, 1.78)
Social frailty	20.78 (17.06, 25.28)	2.10 (1.67, 2.65)
Per score increase	/	1.33 (1.24, 1.43)
**Domains of social frailty**		
General resources	17.56 (15.42, 19.99)	1.57 (1.32, 1.87)
Social behaviors	15.52 (13.91, 17.32)	1.41 (1.19, 1.67)
Social resources	12.15 (9.62, 15.32)	0.90 (0.70, 1.16)
Basic social needs	14.76 (12.60, 17.28)	1.42 (1.18, 1.72)
***One of the conditions to multimorbidity***		
**Social frailty**		
No	18.47 (17.10, 19.94)	Ref
Social pre-frailty	24.47 (21.49, 27.85)	1.23 (1.06, 1.44)
Social frailty	23.43 (19.46, 28.16)	1.23 (1.00, 1.51)
Per score increase	/	1.10 (1.04, 1.17)
**Domains of social frailty**		
General resources	26.08 (23.45, 28.98)	1.19 (1.04, 1.37)
Social behaviors	22.03 (20.10, 24.15)	1.06 (0.93, 1.20)
Social resources	21.84 (18.39, 25.92)	1.07 (0.88, 1.30)
Basic social needs	21.12 (18.52, 24.07)	1.13 (0.97, 1.32)

*HR *hazard ratio, *CI *confidence interval. Social frailty is measured by four domains: general resources (scored 0–1), social behaviors (scored 0–1), social resources (scored 0–1), and basic social needs (scored 0–1); higher score of each domain means higher degree of frailty. Summed scores of 2 and > = 3 from four domains (general resources, social behaviors, social resources, and basic social needs) are then set as social pre-frailty and social frailty, respectively. Models were adjusted for age, sex, study, alcohol consumption, smoking status, and body mass index

Multi-state models specifying the first occurring conditions showed that 2582 (36.3%), 727 (10.2%), and 670 (9.4%) participants transitioned from no physical, psychological, or cognitive conditions to the physical, psychological, and cognitive conditions, respectively. From these individual conditions, 448 (17.4%), 296 (40.7%), and 254 (37.9%) further transitioned to multimorbidity (Fig. 1b). The association between social frailty and transitions from no physical, psychological, or cognitive conditions to the first occurring conditions still existed, with HRs of 1.24 (95% CI = 1.09 to 1.41), 1.49 (95% CI = 1.18 to 1.90), and 1.34 (95% CI = 1.02 to 1.75) for transitioning to physical, psychological, and cognitive conditions, respectively. Social frailty was associated with the transition from the physical condition to multimorbidity (HR = 1.36, 95% CI = 1.02 to 1.83), but not those from other individual conditions. With each additional score of social frailty, the risk of this transition rose 15% (HR = 1.15, 95% CI = 1.05 to 1.25, Additional file 1: Table S4).

The incidence rates of all the abovementioned transitions were higher in participants reporting frailty in general resources than those reporting frailty in other social frailty domains (e.g., 17.56, 95% CI = 15.42 to 19.99 per 1000 person-years of transitioning from no physical, psychological, or cognitive conditions to multimorbidity). Except for social resources, all domains of social frailty were associations with transitions from no physical, psychological, or cognitive conditions to one of the conditions and multimorbidity (e.g., HR = 1.57, 95% CI = 1.32 to 1.87 for frailty in general resources in transitioning from no physical, psychological, or cognitive condition to multimorbidity). When specifying the first occurring conditions, frailty in general resources was associated with higher risks of transitions from no physical, psychological, or cognitive conditions to the physical (HR = 1.16, 95% CI = 1.06 to 1.26) and cognitive conditions (HR = 1.44, 95% CI = 1.21 to 1.70), while no association was observed between social frailty domains and transitions from individual conditions to multimorbidity. The association of each measurement of social frailty domains with multimorbidity transitions exhibited similar results (Additional file 1: Table S5). No differences in these associations were observed by age and sex (Additional file 1: Table S6), and the pooled HRs for transitions from no physical, psychological, or cognitive conditions to multimorbidity were also statistically significant, with no between-study heterogeneity (Additional file 1: Table S7).

### Trajectories of physical, psychological, and cognitive conditions

**Fig. 2:**
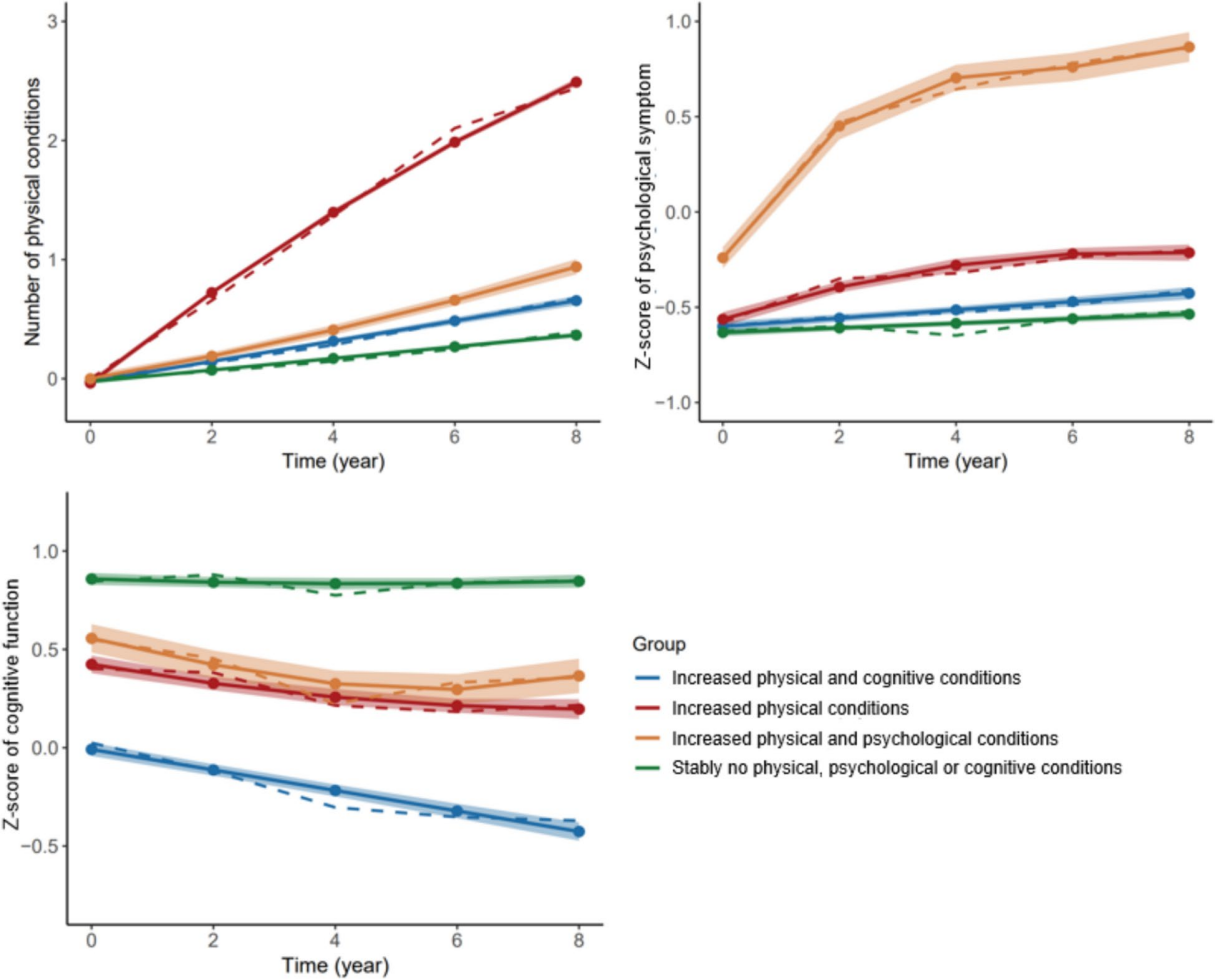
Multi-trajectories of physical, psychological, and cognitive conditions

Four distinct trajectory groups of physical, psychological, and cognitive conditions were identified in 5382 participants (Additional file 1: Table S8, Fig. 2). The groups of “stably no physical, psychological or cognitive conditions” (group 1, reference), “increased physical conditions” (group 2, rapid growth of physical conditions), “increased physical and psychological conditions” (group 3, steady growth of physical conditions and increase in psychological symptoms), and “increased physical and cognitive conditions” (group 4, steady growth of physical conditions and cognitive decline) comprised 2585 (48.0%), 882 (16.4%), 469 (8.7%), and 1446 (26.9%) participants, respectively.

The baseline characteristics of participants according to trajectories of physical, psychological, and cognitive conditions were presented in Additional file 1: Table S9. After adjusting covariates, regarding the “stably no physical, psychological or cognitive conditions” group as reference, social pre-frailty and social frailty were associated with all the remaining trajectory groups, with highest ORs for the “increased physical and cognitive conditions” group (social pre-frailty vs. no social frailty: OR = 2.06, 95% CI = 1.73 to 2.45; social frailty: OR = 2.69, 95% CI = 2.11 to 3.44, Table 3). With each additional score of social frailty, the odds rose 43% (OR = 1.43, 95% CI = 1.32 to 1.55) for the “increased physical conditions” group, 42% (OR = 1.42, 95% CI = 1.29 to 1.56) for the “increased physical and psychological conditions” group, and 54% (OR = 1.54, 95% CI = 1.43 to 1.65) for the “increased physical and cognitive conditions” group. Domains of social frailty except social resources also showed significant associations with all these trajectory groups (e.g., frailty in basic social needs and the “increased physical and psychological conditions” group: OR = 2.27, 95% CI = 1.90 to 2.97). The association of each measurement of social frailty domains with trajectory groups exhibited similar results (Additional file 1: Table S10). Imputing missing values in outcomes yielded a sample size of 7119 participants and broadly similar results of the association between social frailty and trajectories of physical, psychological, and cognitive conditions to those in the main analyses (Additional file 1: Fig. S1 and Table S11). The association between social frailty and the “increased physical and psychological conditions” group were more pronounced in adults aged < 65 years, while no variations of the association were observed by sex (Additional file 1: Tables S12, S13). In random-effects meta-analyses, the association between social frailty between trajectory groups of physical, psychological, and cognitive conditions still existed, with nonsignificant between-study heterogeneity (Additional file 1: Table S14).

**Table 3:** The association between social frailty and different trajectories of physical, psychological, and cognitive conditions

	Increased physical conditions (*n* = 882)		Increased physical and psychological conditions (*n* = 469)		Increased physical and cognitive conditions (*n* = 1446)	
	Events (%)	OR (95% CI)	Events (%)	OR (95% CI)	Events (%)	OR (95% CI)
**Social frailty**						
No	575 (15.0)	Ref	304 (7.9)	Ref	907 (23.6)	Ref
Social pre-frailty	200 (19.5)	1.80 (1.47, 2.21)	104 (10.2)	1.74 (1.35, 2.25)	351 (34.2)	2.06 (1.73, 2.45)
Social frailty	107 (20.9)	2.34 (1.76, 3.09)	61 (11.9)	2.27 (1.63, 3.15)	188 (36.7)	2.69 (2.11, 3.44)
Per score increase	/	1.43 (1.32, 1.55)	/	1.42 (1.29, 1.56)	/	1.54 (1.43, 1.65)
**Domains of social frailty**						
General resources	302 (21.7)	2.12 (1.77–2.55)	137 (9.9)	1.74 (1.38–2.18)	504 (36.2)	2.32 (1.98–2.72)
Social behaviors	409 (18.9)	1.55 (1.31–1.83)	208 (9.6)	1.44 (1.17–1.77)	685 (31.6)	1.89 (1.63–2.18)
Social resources	119 (17.4)	1.15 (0.90–1.47)	57 (8.4)	0.85 (0.62–1.16)	196 (28.7)	0.98 (0.79–1.20)
Basic social needs	210 (17.8)	1.61 (1.32–1.96)	156 (13.2)	2.37 (1.90–2.97)	351 (29.8)	1.50 (1.27–1.78)

*OR *odds ratio, *CI *confidence interval. Social frailty is measured by four domains: general resources (scored 0–1), social behaviors (scored 0–1), social resources (scored 0–1), and basic social needs (scored 0–1); higher score of each domain means higher degree of frailty. Summed scores of 2 and > = 3 from four domains (general resources, social behaviors, social resources, and basic social needs) are then set as social pre-frailty and social frailty, respectively. Models were adjusted for age, sex, study, alcohol consumption, smoking status, and body mass index

## Discussion

This study used harmonized data from 7119 middle-aged and older adults from four aging cohorts across 18 countries and found that social frailty was associated with a higher incidence and risk of developing physical, psychological, and cognitive conditions, as well as multimorbidity. With the exception of social resources, these associations were observed for all domains of social frailty. Among those living with a condition, social frailty was related to faster transitions from a physical condition to multimorbidity but not those from a psychological or cognitive condition to multimorbidity. Trajectory analyses additionally linked social frailty to trajectories of “increased physical conditions,” “increased physical and psychological conditions,” and “increased physical and cognitive conditions.” Although the association with the first trajectory was more pronounced in adults aged < 65 years, no other differences were observed by sex or cohort.

Our findings add to previous evidence on social frailty. They confirm investigations which separately studied the associations of social frailty with physical, psychological, and cognitive outcomes [7, 9–12, 34, 35]. Unlike these studies, we examined these conditions and their multimorbidity in a single analytic setting controlling for death as a competing risk event. Multidimensional outcomes in relation to social frailty have been assessed only in a handful of studies [20, 21]. To our knowledge, the present study is also the first to examine the temporal sequence between physical, psychological, and cognitive conditions, as well as their progression to multimorbidity. This allowed us to disentangle the role of social frailty in each health transition. Compared to no social frailty, we found that both social pre-frailty and social frailty were linked to increased outcomes, but the associations were stronger for social frailty, supporting dose–response pattern in risk.

Among those with no conditions, social frailty appeared to contribute to transitioning to all of the physical, psychological, and cognitive conditions. For participants already with one of the physical, psychological, and cognitive conditions, social frailty contributed to the transition from a physical condition to multimorbidity with psychological or cognitive comorbidities, but not transitions from psychological or cognitive conditions to multimorbidity. The null association from the psychological or cognitive conditions might be partially explained by the small sample size, as only 296 and 254 participants transitioned to multimorbidity from these conditions. In addition, as cognitive impairment is a long-term progressive condition, we might miss many cases of cognitive decline in the limited,on average, 7-year follow-up. Future studies with larger sample size and longer follow-up periods are warranted to confirm and complement our findings.

To comprehensively evaluate social frailty in middle-aged and older adults across various countries, four distinct domains were captured including general resources, social behaviors, social resources, and basic social needs. Except social resources, all domains of social frailty presented were associated with faster transitions to physical, psychological, and cognitive conditions and multimorbidity. The social production function theory suggests that social wellbeing of a person depends on his/her ability to fulfill the following three social needs: affection, behavioral confirmation, and status [3, 36]. In our analysis, the level of general resources (educational level and total household wealth), social behaviors (monthly social activities and employment), and basic social needs (satisfaction with life and weekly contact with children) may be more dependent on individuals’ initiative than that of social resources (marital status and number of living children), potentially explaining the stronger associations with physical, psychological, and cognitive health. Further research is needed to examine this hypothesis, including possible context-specific differences.

The progression of psychological and cognitive conditions and multimorbidity at older ages is a fluctuating and long-term process. Therefore, we conducted GBMTM to identify distinct trajectories in terms of the number of the physical conditions, psychological symptoms, and cognitive function, including those which might not have reached the thresholds of psychological and cognitive conditions. Four trajectory groups were identified, with more than half of participants showing an increase in physical conditions, heightened psychological symptoms, or a decline in cognitive function. Social pre-frailty and social frailty contributed to these trajectories, particularly the decline in cognitive function. Previous evidence has shown social frailty to be associated with an acceleration in the development of depressive symptoms and a faster cognitive decline [10, 11, 20, 21], whereas our study also emphasizes the accumulation of chronic physical conditions in relation to social frailty. Subgroup analysis presented that the association between social frailty and the trajectories of physical, psychological, and cognitive conditions was most pronounced among middle-aged adults. Previous evidence has established age as a significant predictor of social frailty, with the prevalence of social frailty elevating with age.

### Clinical and public health implications

These findings provide the predictive ability of social frailty for risk of complex multimorbidity involving physical, psychological, and cognitive conditions, having important implications in terms of prevention and clinical practice.

On one hand, social frailty may facilitate early identification of individuals at higher risk of chronic conditions and multimorbidity and the timely implementation prevention strategies, even if they have occurred one condition. Health professionals should raise awareness of social frailty and closely monitor individuals affected by social frailty to deliver tailor clinical interventions and prevent deterioration of health. Guided by the social-ecological model [37], social connection can be integrated into primary-, secondary-, and tertiary-level care to strengthen patient social robustness.

On the other hand, promoting social behavioral activities, like maintaining relationships and social participation, would help individuals with social frailty achieve the goal of fulfilling social needs [30] and therefore delay or prevent downstream health deterioration [38]. Health education of self-management (e.g., cognitive behavioral therapy, skills training) would improve an individual’s ability to manage their behaviors, emotions, and lifestyle [39], which is also a prominent intervention to both address social frailty and improve overall health [38, 40].

### Limitations, strengths, future studies

The strengths of our study include the use of harmonized data from four aging cohorts across 18 countries, a comprehensive assessment of social frailty, and the use of multi-state models and GBDTM to examine the development of physical, psychological, and cognitive conditions and multimorbidity. However, this study also has several limitations. First, social frailty was measured using self-reported information, which is subject to recall and social desirability biases [41]. Due to data unavailability and limited sample size, we failed to further specify the aspects of basic social needs, and future studies with more social frailty indicators and larger sample size are warranted. In addition, data on physical conditions were also self-reported and thus therefore susceptible to misclassification. However, previous studies have demonstrated good validity of self-reported information on chronic physical conditions [42–44]. Second, harmonized data were only available for seven common chronic physical conditions. Consequently, we may have missed many conditions, reducing the ability to examine a wider range of transitions between health conditions and multimorbidity. Third, the psychological condition referred to depressive symptoms, and information on other psychological conditions was unavailable. Future studies including more psychological conditions (e.g., schizophrenia, anxiety) are warranted. In addition, we relied on predefined study-specific instruments to measure psychological and cognitive conditions. Although these instruments have been validated in local populations, heterogeneity in the cohort-specific results may exist. Despite this, few cohort-specific differences were observed across the studies. Fourth, the measurement of social frailty varied between studies, potentially increasing heterogeneity and influencing the strength of the associations. Future research using studies with standardized definition and measurement could provide more precise estimates of the role of social frailty in the development of physical, psychological, and cognitive conditions and multimorbidity. Fifth, the mean follow-up of this study was too short to observe advanced multimorbidity developing over decades, contributing to an underestimation of the associations. Results from GBMTM revealed that social frailty also predicted the adverse changes of physical, psychological, and cognitive conditions. Therefore, more prospective studies with longer follow-ups are warranted to validate our findings and further explore the underlying mechanisms. In addition, the follow-ups were conducted every 2 to 3 years, not necessarily capturing all the temporal transitions to physical, psychological, and cognitive conditions. Finally, the participants included were from high-income countries or upper-middle-income countries. Whether the findings are generalizable to populations in lower-income countries warrants further verification.

## Conclusions

Social frailty was identified as a risk factor for physical, psychological, and cognitive conditions, as well as for progression to multimorbidity. Health professionals should raise awareness of social frailty and closely monitor individuals affected by social frailty to deliver tailor clinical interventions and prevent deterioration of health. Public health efforts, including promoting social behavioral activities and health education of self-management, would also help delay or prevent downstream health deterioration in individuals with social frailty.

## Supplementary Information

Additional file 1. Table S1-S14. Table S1 – Characteristics of studies included in present analyses. Table S2 – Harmonized strategies for key variables. Table S3 – Baseline characteristics of included participants. Table S4 – The association between social frailty and multimorbidity (each condition specified). Table S5 – The association between each measurement of social frailty domains and multimorbidity. Table S6 – Subgroup analyses for multi-state models by age and sex. Table S7 – Subgroup analyses for multi-state models by study. Table S8 – Model selection for the multi-trajectory modelling. Table S9 – Baseline characteristics of included participants in the trajectory analysis. Table S10 – The association between each measurement of social frailty domains and different trajectories. Table S11 – The association between social frailty and different trajectories after multiple imputation. Table S12 – Subgroup analyses for trajectory analysis by age. Table S13 – Subgroup analyses for trajectory analysis by sex. Table S14 – Meta-analysis for trajectory analysis. Figure S1 – Multi-trajectories after multiple imputation.

## Data Availability

The original data for this study are freely available on their respective websites: The Health and Retirement Study-HRS (https://hrs.isr.umich.edu/), the English Longitudinal Study of Ageing-ELSA (https://www.elsa-project.ac.uk), the Survey of Health, Ageing and Retirement in Europe-SHARE (http://www.share-project.org/home0.html), and the China Health and Retirement Longitudinal Study-CHARLS (http://charls.pku.edu.cn/index/en.html). Access can be obtained after registration.

## Abbreviations

AIC: Akaike information criterion
BIC: Bayesian information criteria
BMI: Body mass index
CESD: The Center for Epidemiologic Studies Depression Scale
CHARLS: China Health and Retirement Longitudinal Study
CI: Confidence interval
ELSA: English Longitudinal Study on Ageing
GBMTM: Group-based multi-trajectory model
HR: Hazard ratio
HRS: US Health and Retirement Study
OR: Odds ratios
SD: Standard deviation
SHARE: Survey of Health, Ageing and Retirement in Europe
SPF: Social production function
STROBE: Strengthening the Reporting of Observational Studies in Epidemiology
