# Supplementary material for: Social frailty and the risk of progression from health to physical, psychological, and cognitive multimorbidity: a prospective multi-cohort study
Source: BMC Med. 2026 Mar 6;24:240. doi: 10.1186/s12916-026-04761-8 (PMC13077994; doi:10.1186/s12916-026-04761-8)
Supplement: Supplementary file 1 — Additional file 1. Table S1-S14. Table S1 – Characteristics of studies included in present analyses. Table S2 – Harmonized strategies for key variables. Table S3 – Baseline characteristics of included participants. Table S4 – The association between social frailty and multimorbidity (each condition specified). Table S5 – The association between each measurement of social frailty domains and multimorbidity. Table S6 – Subgroup analyses for multi-state models by age and sex. Table S7 – Subgroup analyses for multi-state models by study. Table S8 – Model selection for the multi-trajectory modelling. Table S9 – Baseline characteristics of included participants in the trajectory analysis. Table S10 – The association between each measurement of social frailty domains and different trajectories. Table S11 – The association between social frailty and different trajectories after multiple imputation. Table S12 – Subgroup analyses for trajectory analysis by age. Table S13 – Subgroup analyses for trajectory analysis by sex. Table S14 – Meta-analysis for trajectory analysis. Figure S1 – Multi-trajectories after multiple imputation. [file 12916_2026_4761_MOESM1_ESM.docx]

**Social frailty and the risk of progression from health to physical, psychological and cognitive multimorbidity: a prospective multi-cohort study**

**Supplementary materials**

**Table S1**. Characteristics of studies included in present analyses.

**Table S2**. Harmonized strategies for key variables included in present analyses.

**Table S3**. Baseline characteristics of included participants according to the development of physical, psychological and cognitive multimorbidities (n=7,119).

**Table S4**. Incidence rates and hazard ratios for the association between social frailty and the progression of physical, psychological and cognitive multimorbidities (each condition specified).

**Table S5**. Incidence rates and hazard ratios for the association between each measurement of social frailty domains and progressions of physical, psychological and cognitive multimorbidities.

**Table S6**. Multi-state models for the association between social frailty and the progression of physical, psychological and cognitive multimorbidities by age and sex.

**Table S7**. Meta-analysis for the association between social frailty and the progression of physical, psychological and cognitive multimorbidities by study.

**Table S8.** Model selection for the multi-trajectories of physical, psychological and cognitive conditions.

**Table S9**. Baseline characteristics of included participants according to the trajectory of physical, psychological and cognitive conditions (n=5,382).

**Table S10**. The association between each measurement of social frailty domains and different trajectories of physical, psychological and cognitive conditions.

**Table S11**. The association between social frailty and different trajectories of physical, psychological and cognitive conditions after multiple imputation.

**Table S12**. The association between social frailty and different trajectories of physical, psychological and cognitive conditions by age.

**Table S13.** The association between social frailty and different trajectories of physical, psychological and cognitive conditions by sex.

**Table S14**. Meta-analysis for the association between social frailty and different trajectories of physical, psychological and cognitive conditions by study.

**Figure S1**. Multi-trajectories of physical, psychological and cognitive conditions after multiple imputation (N=7,119).

**Table S1**. Characteristics of studies included in present analyses.

| Study | Country | Wave | Time period | Total | Mean age (SD) | Female (%) |
| --- | --- | --- | --- | --- | --- | --- |
| HRS | US | 11-15 | 2012-2021 | 171 | 71.49 (6.10) | 94 (54.97) |
| ELSA | England | 5-9 | 2010-2019 | 1272 | 62.88 (7.12) | 680 (53.46) |
| SHARE | Austria | 5-9 | 2012-2021 | 43 | 61.60 (8.72) | 19 (44.19) |
|  | Belgium | 5-9 | 2012-2021 | 284 | 58.42 (8.75) | 139 (48.94) |
|  | Czech Republic | 5-9 | 2012-2021 | 253 | 60.22 (7.04) | 140 (55.34) |
|  | Denmark | 5-9 | 2012-2021 | 481 | 59.81 (8.82) | 261 (54.26) |
|  | Estonia | 5-9 | 2012-2021 | 36 | 59.69 (7.32) | 15 (41.67) |
|  | France | 5-9 | 2012-2021 | 32 | 58.72 (8.19) | 19 (59.38) |
|  | Germany | 5-9 | 2012-2021 | 768 | 58.49 (7.97) | 417 (54.30) |
|  | Israel | 5-9 | 2012-2021 | 137 | 56.20 (5.50) | 76 (55.47) |
|  | Italy | 5-9 | 2012-2021 | 349 | 58.74 (7.97) | 179 (51.29) |
|  | Luxembourg | 5-9 | 2012-2021 | 201 | 60.09 (7.74) | 103 (51.24) |
|  | Netherlands | 5-9 | 2012-2021 | 286 | 60.16 (7.63) | 146 (51.05) |
|  | Slovenia | 5-9 | 2012-2021 | 202 | 59.49 (8.10) | 125 (61.88) |
|  | Spain | 5-9 | 2012-2021 | 494 | 60.35 (8.25) | 254 (51.42) |
|  | Sweden | 5-9 | 2012-2021 | 587 | 62.32 (8.35) | 289 (49.23) |
|  | Switzerland | 5-9 | 2012-2021 | 23 | 61.34 (6.52) | 12 (52.17) |
| CHARLS | China | 1-5 | 2011-2021 | 1500 | 55.21 (7.85) | 607 (40.47) |
| Total | / | / | / | 7119 | 59.63 (8.45) | 3575 (50.22) |

SD, standard deviation; HRS, US Health and Retirement Study; ELSA, English Longitudinal Study on Ageing; SHARE, Survey of Health, Ageing and Retirement in Europe; CHARLS, China Health and Retirement Longitudinal Study.

**Table S2**. Harmonized strategies for key variables included in present analyses.

| **Variables** | **Harmonized values** | **Measurements in three studies** | | | |
| --- | --- | --- | --- | --- | --- |
|  |  | **HRS** | **ELSA** | **SHARE** | **CHARLS** |
| Social frailty | Social frailty | Summed score of >=3 from four domains (general resources, social behaviors, social resources and basic social needs) | | | |
|  | Social pre-frailty | Summed score of 2 from four domains (general resources, social behaviors, social resources and basic social needs) | | | |
|  | No | Summed score of 0-1 from four domains (general resources, social behaviors, social resources and basic social needs) | | | |
| General resources | Score 0 | In the first tertile of either total household wealth or educational level. | | | |
|  | Score 1 | Otherwise. | | | |
| Social behaviors | Score 0 | Reported no monthly social activities or unemployment. | | | |
|  | Score 1 | Otherwise. | | | |
| Social resources | Score 0 | Not married/partnered or having no living children. | | | |
|  | Score 1 | Otherwise. | | | |
| Basic social needs | Score 0 | Lower than the mean score of satisfaction with life or no weekly contact with children. | | | |
|  | Score 1 | Otherwise. | | | |
| Physical condition | Yes | Reported any of hypertension, diabetes, stroke, heart problems, arthritis, lung disease, cancer. | | | |
|  | No | Did not report above mentioned physical chronic condition. | | | |
| Psychological condition | Yes | CESD-8 score ≥4 | CESD-8 score ≥4 | EURO-D score ≥4 | CESD-10 score ≥10 |
|  | No | CESD-8 score <4 | CESD-8 score <4 | EURO-D score <4 | CESD-10 score <10 |
| Cognitive condition | Yes | Having a Z-score >1.5 below means in at least one of four cognitive domains: immediate word recall, delayed word recall, serial 7’s subtraction test, and orientation | Having a Z-score >1.5 below means in at least one of four cognitive domains: immediate word recall, delayed word recall, animal  fluency test, and orientation | Having a Z-score >1.5 below means in at least one of four cognitive domains: immediate word recall, delayed word recall, serial 7’s subtraction test, and orientation | Having a Z-score >1.5 below means in at least one of five cognitive domains: immediate word recall, delayed word recall, serial 7’s subtraction test, and orientation |
|  | No | Otherwise | Otherwise | Otherwise | Otherwise |

HRS, US Health and Retirement Study; ELSA, English Longitudinal Study on Ageing; SHARE, Survey of Health, Ageing and Retirement in Europe; CHARLS, China Health and Retirement Longitudinal Study; CESD, Center for Epidemiological Studies Depression.

**Table S3**. Baseline characteristics of included participants according to the development of physical, psychological and cognitive multimorbidities (n=7119).

|  | Total (n=7119) | The development of physical, psychological and cognitive multimorbidities | | | | | p-value |
| --- | --- | --- | --- | --- | --- | --- | --- |
|  |  | None (n=7119) | Physical conditions (n=2134) | Psychological condition (n=431) | Cognitive condition (n=416) | Multimorbidities (n=1592) |  |
| **Age group (years)** |  |  |  |  |  |  | <0.001 |
| 45-54 | 2228 (31.3) | 880 (39.5) | 542 (24.3) | 197 (8.8) | 119 (5.3) | 490 (22.0) |  |
| 55-64 | 2968 (41.7) | 1136 (38.3) | 924 (31.1) | 176 (5.9) | 155 (5.2) | 577 (19.4) |  |
| 65-74 | 1519 (21.3) | 449 (29.6) | 553 (36.4) | 47 (3.1) | 103 (6.8) | 367 (24.2) |  |
| 75- | 404 (5.7) | 81 (20.0) | 115 (28.5) | 11 (2.7) | 39 (9.7) | 158 (39.1) |  |
| **Sex** |  |  |  |  |  |  | <0.001 |
| Man | 3544 (49.8) | 1199 (33.8) | 1181 (33.3) | 152 (4.3) | 238 (6.7) | 774 (21.8) |  |
| Woman | 3575 (50.2) | 1347 (37.7) | 953 (26.7) | 279 (7.8) | 178 (5.0) | 818 (22.9) |  |
| **Current smoking** |  |  |  |  |  |  | <0.001 |
| No | 5646 (79.3) | 2085 (36.9) | 1720 (30.5) | 338 (6.0) | 309 (5.5) | 1194 (21.1) |  |
| Yes | 1473 (20.7) | 461 (31.3) | 414 (28.1) | 93 (6.3) | 107 (7.3) | 398 (27.0) |  |
| **Alcohol consumption** |  |  |  |  |  |  | <0.001 |
| Less than weekly drinking | 3380 (47.5) | 1083 (32.0) | 906 (26.8) | 217 (6.4) | 218 (6.4) | 956 (28.3) |  |
| Weekly drinking or more | 3739 (52.5) | 1463 (39.1) | 1228 (32.8) | 214 (5.7) | 198 (5.3) | 636 (17.0) |  |
| **Body mass index (kg/m2)** |  |  |  |  |  |  | <0.001 |
| <18.5 | 117 (1.6) | 34 (29.1) | 20 (17.1) | 8 (6.8) | 12 (10.3) | 43 (36.8) |  |
| 18.5-23.9 | 3495 (49.1) | 1309 (37.5) | 900 (25.8) | 249 (7.1) | 225 (6.4) | 812 (23.2) |  |
| 24.0-29.9 | 2738 (38.5) | 981 (35.8) | 913 (33.3) | 129 (4.7) | 143 (5.2) | 572 (20.9) |  |
| 30.0- | 769 (10.8) | 222 (28.9) | 301 (39.1) | 45 (5.9) | 36 (4.7) | 165 (21.5) |  |
| **Social frailty** |  |  |  |  |  |  | <0.001 |
| No | 4937 (69.3) | 1878 (38.0) | 1473 (29.8) | 281 (5.7) | 297 (6.0) | 1008 (20.4) |  |
| Social pre-frailty | 1404 (19.7) | 437 (31.1) | 423 (30.1) | 102 (7.3) | 75 (5.3) | 367 (26.1) |  |
| Social frailty | 778 (10.9) | 231 (29.7) | 238 (30.6) | 48 (6.2) | 44 (5.7) | 217 (27.9) |  |
| **General resources** |  |  |  |  |  |  | <0.001 |
| No frailty | 5131 (72.1) | 1940 (37.8) | 1555 (30.3) | 322 (6.3) | 296 (5.8) | 1018 (19.8) |  |
| Frailty | 1988 (27.9) | 606 (30.5) | 579 (29.1) | 109 (5.5) | 120 (6.0) | 574 (28.9) |  |
| **Social behaviors** |  |  |  |  |  |  | <0.001 |
| No frailty | 4019 (56.5) | 1532 (38.1) | 1193 (29.7) | 235 (5.8) | 246 (6.1) | 813 (20.2) |  |
| Frailty | 3100 (43.5) | 1014 (32.7) | 941 (30.4) | 196 (6.3) | 170 (5.5) | 779 (25.1) |  |
| **Social resources** |  |  |  |  |  |  | 0.413 |
| No frailty | 6246 (87.7) | 2259 (36.2) | 1865 (29.9) | 377 (6.0) | 360 (5.8) | 1385 (22.2) |  |
| Frailty | 873 (12.3) | 287 (32.9) | 269 (30.8) | 54 (6.2) | 56 (6.4) | 207 (23.7) |  |
| **Basic social needs** |  |  |  |  |  |  | <0.001 |
| No frailty | 5520 (77.5) | 2036 (36.9) | 1632 (29.6) | 310 (5.6) | 334 (6.1) | 1208 (21.9) |  |
| Frailty | 1599 (22.5) | 510 (31.9) | 502 (31.4) | 121 (7.6) | 82 (5.1) | 384 (24.0) |  |

Social frailty is measured by four domains: general resources (scored 0-1), social behaviors (scored 0-1), social resources (scored 0-1) and basic social needs (scored 0-1); higher score of each domain means higher degree of frailty. Summed scores of 2 and >=3 from four domains (general resources, social behaviors, social resources and basic social needs) are then set as social pre-frailty and social frailty, respectively.

**Table S4**. Incidence rates and hazard ratios for the association between social frailty and the progression of physical, psychological and cognitive multimorbidities (each condition specified).

|  | Incidence rate per 1000 person-years | HR (95% CI) |
| --- | --- | --- |
| ***N to P_1_*** |  |  |
| **Social frailty** |  |  |
| No | 49.83 (47.59, 52.16) | Ref |
| Social pre-frailty | 55.62 (51.12, 60.48) | 1.16 (1.05, 1.28) |
| Social frailty | 60.72 (54.28, 67.86) | 1.24 (1.09, 1.41) |
| Per score increase | / | 1.09 (1.05, 1.13) |
| **Domains of social frailty** |  |  |
| General resources | 56.18 (52.34, 60.29) | 1.16 (1.06, 1.26) |
| Social behaviors | 55.97 (52.90, 59.21) | 1.14 (1.05, 1.24) |
| Social resources | 54.36 (48.86, 60.43) | 0.98 (0.87, 1.10) |
| Basic social needs | 56.72 (52.45, 61.31) | 1.11 (1.01, 1.22) |
| ***N to P_2_*** |  |  |
| **Social frailty** |  |  |
| No | 13.46 (12.29, 14.73) | Ref |
| Social pre-frailty | 17.80 (15.27, 20.73) | 1.51 (1.26, 1.80) |
| Social frailty | 17.11 (13.75, 21.25) | 1.49 (1.18, 1.90) |
| Per score increase | / | 1.16 (1.08, 1.24) |
| **Domains of social frailty** |  |  |
| General resources | 15.13 (13.15, 17.40) | 1.07 (0.90, 1.27) |
| Social behaviors | 15.77 (14.13, 17.58) | 1.04 (0.89, 1.21) |
| Social resources | 15.60 (12.71, 19.12) | 1.16 (0.92, 1.45) |
| Basic social needs | 18.31 (15.90, 21.08) | 1.62 (1.36, 1.92) |
| ***N to C*** |  |  |
| **Social frailty** |  |  |
| No | 13.29 (12.13, 14.55) | Ref |
| Social pre-frailty | 14.62 (12.34, 17.30) | 1.31 (1.08, 1.58) |
| Social frailty | 12.84 (9.96, 16.50) | 1.34 (1.02, 1.75) |
| Per score increase | / | 1.17 (1.08, 1.26) |
| **Domains of social frailty** |  |  |
| General resources | 16.19 (14.14, 18.54) | 1.44 (1.21, 1.70) |
| Social behaviors | 13.31 (11.81, 14.98) | 1.10 (0.94, 1.29) |
| Social resources | 14.12 (11.38, 17.50) | 1.04 (0.82, 1.32) |
| Basic social needs | 11.96 (10.03, 14.25) | 0.99 (0.81, 1.21) |
| ***N to PPC-MM*** |  |  |
| **Social frailty** |  |  |
| No | 10.08 (9.08, 11.20) | Ref |
| Social pre-frailty | 14.41 (12.15, 17.07) | 1.45 (1.19, 1.78) |
| Social frailty | 20.78 (17.06, 25.28) | 2.10 (1.67, 2.65) |
| Per score increase | / | 1.33 (1.24, 1.43) |
| **Domains of social frailty** |  |  |
| General resources | 17.56 (15.42, 19.99) | 1.57 (1.32, 1.87) |
| Social behaviors | 15.52 (13.91, 17.32) | 1.41 (1.19, 1.67) |
| Social resources | 12.15 (9.62, 15.32) | 0.90 (0.70, 1.16) |
| Basic social needs | 14.76 (12.60, 17.28) | 1.42 (1.18, 1.72) |
| ***P_1_ to PPC-MM*** |  |  |
| **Social frailty** |  |  |
| No | 8.10 (7.21, 9.11) | Ref |
| Social pre-frailty | 10.81 (8.86, 13.16) | 1.20 (0.95, 1.51) |
| Social frailty | 12.22 (9.42, 15.82) | 1.36 (1.02, 1.83) |
| Per score increase | / | 1.15 (1.05, 1.25) |
| **Domains of social frailty** |  |  |
| General resources | 12.16 (10.39, 14.23) | 1.21 (0.99, 1.49) |
| Social behaviors | 10.61 (9.28, 12.12) | 1.17 (0.96, 1.42) |
| Social resources | 10.18 (7.88, 13.12) | 1.08 (0.80, 1.44) |
| Basic social needs | 9.81 (8.07, 11.91) | 1.09 (0.86, 1.37) |
| ***P_2_ to PPC-MM*** |  |  |
| **Social frailty** |  |  |
| No | 5.50 (4.76, 6.34) | Ref |
| Social pre-frailty | 6.99 (5.45, 8.94) | 1.12 (0.84, 1.49) |
| Social frailty | 7.33 (5.22, 10.26) | 1.09 (0.75, 1.58) |
| Per score increase | / | 1.06 (0.95, 1.19) |
| **Domains of social frailty** |  |  |
| General resources | 6.84 (5.54, 8.44) | 1.29 (0.99, 1.68) |
| Social behaviors | 6.32 (5.30, 7.52) | 0.95 (0.75, 1.21) |
| Social resources | 6.73 (4.90, 9.22) | 1.06 (0.74, 1.52) |
| Basic social needs | 7.01 (5.55, 8.83) | 1.02 (0.78, 1.34) |
| ***C to PPC-MM*** |  |  |
| **Social frailty** |  |  |
| No | 4.87 (4.18, 5.67) | Ref |
| Social pre-frailty | 6.67 (5.17, 8.59) | 1.22 (0.90, 1.65) |
| Social frailty | 3.87 (2.40, 6.16) | 0.90 (0.55, 1.46) |
| Per score increase | / | 1.03 (0.90, 1.17) |
| **Domains of social frailty** |  |  |
| General resources | 7.07 (5.74, 8.70) | 1.06 (0.81, 1.39) |
| Social behaviors | 5.11 (4.21, 6.20) | 1.05 (0.81, 1.36) |
| Social resources | 4.93 (3.39, 7.12) | 0.98 (0.66, 1.47) |
| Basic social needs | 4.30 (3.18, 5.78) | 1.12 (0.81, 1.56) |

N, no physical, psychological or cognitive conditions; P_1_, physical condition; P_2_, psychological condition; C, cognitive condition; MM, multimorbidity. HR, hazard ratio; CI, confidence interval; AF, attributable fractions.

Social frailty is measured by four domains: general resources (scored 0-1), social behaviors (scored 0-1), social resources (scored 0-1) and basic social needs (scored 0-1); higher score of each domain means higher degree of frailty. Summed scores of 2 and >=3 from four domains (general resources, social behaviors, social resources and basic social needs) are then set as social pre-frailty and social frailty, respectively.

Models were adjusted for age, sex, study, alcohol consumption, smoking status and body mass index.

**Table S5**. Incidence rates and hazard ratios for the association between each measurement of social frailty domains and progressions of physical, psychological and cognitive multimorbidities.

|  | Incidence rate per 1000 person-years (95% CI) | HR (95% CI) |
| --- | --- | --- |
| ***No physical, psychological or cognitive conditions to one of the conditions*** | | |
| **General resources** |  |  |
| Low educational level | 84.03 (78.65-89.75) | 1.30 (1.18-1.44) |
| Low total household wealth | 103.46 (94.55-113.09) | 1.08 (1.00-1.17) |
| **Social behaviors** |  |  |
| No monthly social activities | 84.79 (81.02-88.73) | 1.10 (1.03-1.18) |
| Unemployment | 86.57 (69.42-107.34) | 1.08 (0.86-1.35) |
| **Social resources** |  |  |
| Not married/partnered | 84.93 (77.85-92.58) | 1.00 (0.91-1.11) |
| No living children | 75.29 (54.74-102.36) | 1.10 (0.80-1.52) |
| **Basic social needs** |  |  |
| Not satisfied with life | 87.02 (81.19-93.22) | 1.18 (1.09-1.28) |
| No weekly contact with children | 84.75 (74.46-96.29) | 1.04 (0.90-1.19) |
| ***No physical, psychological or cognitive conditions to multimorbidities*** | | |
| **General resources** |  |  |
| Low educational level | 16.36 (13.98-19.12) | 1.57 (1.25-1.98) |
| Low total household wealth | 23.28 (19.05-28.39) | 1.35 (1.12-1.63) |
| **Social behaviors** |  |  |
| No monthly social activities | 15.79 (14.14-17.62) | 1.43 (1.21-1.70) |
| Unemployment | 17.76 (10.54-29.33) | 1.61 (0.97-2.68) |
| **Social resources** |  |  |
| Not married/partnered | 12.77 (10.07-16.14) | 0.90 (0.70-1.17) |
| No living children | 3.86 (0.67-15.45) | 0.66 (0.16-2.68) |
| **Basic social needs** |  |  |
| Not satisfied with life | 15.92 (13.43-18.85) | 1.68 (1.38-2.06) |
| No weekly contact with children | 11.17 (7.63-16.22) | 0.80 (0.55-1.16) |
| ***One of the conditions to multimorbidities*** | | |
| **General resources** |  |  |
| Low educational level | 23.31 (20.45-26.54) | 1.31 (1.09-1.57) |
| Low total household wealth | 35.27 (30.02-41.37) | 1.04 (0.89-1.21) |
| **Social behaviors** |  |  |
| No monthly social activities | 21.94 (19.99-24.07) | 1.04 (0.92-1.19) |
| Unemployment | 19.98 (12.24-32.03) | 1.48 (0.92-2.39) |
| **Social resources** |  |  |
| Not married/partnered | 22.87 (19.20-27.21) | 1.09 (0.89-1.33) |
| No living children | 7.72 (2.48-21.05) | 0.57 (0.21-1.54) |
| **Basic social needs** |  |  |
| Not satisfied with life | 21.38 (18.47-24.72) | 1.28 (1.08-1.52) |
| No weekly contact with children | 20.42 (15.48-26.82) | 0.83 (0.63-1.09) |

HR, hazard ratio; CI, confidence interval.

Models were adjusted for age, sex, study, alcohol consumption, smoking status and body mass index.

**Table S6**. Multi-state models for the association between social frailty and the progression of physical, psychological and cognitive multimorbidities by age and sex.

|  | Hazard ratio (95% confidence interval) | | |
| --- | --- | --- | --- |
|  | No physical, psychological or cognitive conditions to one of the condition | No physical, psychological or cognitive conditions to multimorbidities | One of the conditions to multimorbidities |
| ***<65 years*** |  |  |  |
| No | Ref | Ref | Ref |
| Social pre-frailty | 1.20 (1.08, 1.32) | 1.69 (1.32, 2.16) | 1.35 (1.12, 1.63) |
| Social frailty | 1.37 (1.21, 1.55) | 2.47 (1.83, 3.33) | 1.32 (1.02, 1.72) |
| Per score increase | 1.12 (1.08, 1.16) | 1.41 (1.29, 1.54) | 1.15 (1.07, 1.24) |
| ***>= 65 years*** |  |  |  |
| No | Ref | Ref | Ref |
| Social pre-frailty | 1.36 (1.19, 1.56) | 1.18 (0.83, 1.67) | 1.09 (0.84, 1.41) |
| Social frailty | 1.25 (1.05, 1.49) | 1.95 (1.36, 2.79) | 1.23 (0.88, 1.71) |
| Per score increase | 1.12 (1.06, 1.18) | 1.27 (1.13, 1.44) | 1.07 (0.96, 1.18) |
| ***Male*** |  |  |  |
| No | Ref | Ref | Ref |
| Social pre-frailty | 1.18 (1.06, 1.32) | 1.56 (1.18, 2.05) | 1.22 (0.97, 1.53) |
| Social frailty | 1.27 (1.09, 1.48) | 2.01 (1.41, 2.86) | 1.39 (1.02, 1.90) |
| Per score increase | 1.12 (1.07, 1.16) | 1.34 (1.20, 1.49) | 1.15 (1.05, 1.26) |
| ***Female*** |  |  |  |
| No | Ref | Ref | Ref |
| Social pre-frailty | 1.29 (1.16, 1.45) | 1.35 (1.01, 1.81) | 1.26 (1.02, 1.56) |
| Social frailty | 1.32 (1.14, 1.52) | 2.08 (1.52, 2.85) | 1.15 (0.87, 1.51) |
| Per score increase | 1.11 (1.06, 1.16) | 1.31 (1.18, 1.45) | 1.07 (0.99, 1.16) |

HR, hazard ratio; CI, confidence interval.

Social frailty is measured by four domains: general resources (scored 0-1), social behaviors (scored 0-1), social resources (scored 0-1) and basic social needs (scored 0-1); higher score of each domain means higher degree of frailty. Summed scores of 2 and >=3 from four domains (general resources, social behaviors, social resources and basic social needs) are then set as social pre-frailty and social frailty, respectively.

Models were adjusted for age, sex, study, alcohol consumption, smoking status and body mass index.

**Table S7**. Meta-analysis for the association between social frailty and the progression of physical, psychological and cognitive multimorbidities by study.

|  | No physical, psychological or cognitive conditions to one of the condition | No physical, psychological or cognitive conditions to multimorbidities | One of the conditions to multimorbidities |
| --- | --- | --- | --- |
| ***HRS*** |  |  |  |
| No | Ref | Ref | Ref |
| Social pre-frailty | 1.28 (0.72, 2.28) | 5.11 (0.95, 27.62) | 0.85 (0.28, 2.64) |
| Social frailty | 1.27 (0.64, 2.53) | 2.99 (0.57, 15.72) | 0.89 (0.17, 4.79) |
| Per score increase | 1.14 (0.94, 1.38) | 1.48 (0.91, 2.39) | 1.02 (0.64, 1.61) |
| ***ELSA*** |  |  |  |
| No | Ref | Ref | Ref |
| Social pre-frailty | 1.31 (1.08, 1.60) | 1.48 (0.77, 2.85) | 0.92 (0.59, 1.43) |
| Social frailty | 1.64 (1.28, 2.12) | 0.90 (0.31, 2.62) | 1.51 (0.90, 2.55) |
| Per score increase | 1.18 (1.09, 1.27) | 1.17 (0.9, 1.52) | 1.19 (1.01, 1.4) |
| ***SHARE*** |  |  |  |
| No | Ref | Ref | Ref |
| Social pre-frailty | 1.24 (1.12, 1.38) | 1.37 (1.03, 1.83) | 1.16 (0.92, 1.48) |
| Social frailty | 1.23 (1.08, 1.40) | 2.25 (1.68, 3.01) | 1.18 (0.88, 1.57) |
| Per score increase | 1.10 (1.06, 1.14) | 1.32 (1.20, 1.45) | 1.06 (0.97, 1.16) |
| ***CHARLS*** |  |  |  |
| No | Ref | Ref | Ref |
| Social pre-frailty | 1.13 (0.95, 1.33) | 1.53 (1.11, 2.12) | 1.37 (1.08, 1.72) |
| Social frailty | 1.19 (0.90, 1.58) | 2.10 (1.32, 3.34) | 1.25 (0.85, 1.85) |
| Per score increase | 1.07 (1.00, 1.15) | 1.40 (1.23, 1.60) | 1.15 (1.04, 1.27) |
| ***Pooled*** |  |  |  |
| Social pre-frailty |  |  |  |
| HR (95%CI) | 1.23 (1.13, 1.33) | 1.47 (1.20, 1.80) | 1.20 (1.03, 1.41) |
| Heterogeneity (*I*^2^) | 0% | 0% | 2% |
| Social frailty |  |  |  |
| HR (95%CI) | 1.31 (1.13, 1.53) | 2.12 (1.67, 2.69) | 1.24 (1.01, 1.54) |
| Heterogeneity (*I*^2^) | 30% | 0% | 0% |
| Per score increase |  |  |  |
| HR (95%CI) | 1.11 (1.07, 1.15) | 1.34 (1.24, 1.44) | 1.11 (1.04, 1.18) |
| Heterogeneity (*I*^2^) | 21% | 0% | 0% |

HR, hazard ratio; CI, confidence interval; HRS, US Health and Retirement Study; ELSA, English Longitudinal Study on Ageing; SHARE, Survey of Health, Ageing and Retirement in Europe; CHARLS, China Health and Retirement Longitudinal Study.

Social frailty is measured by four domains: general resources (scored 0-1), social behaviors (scored 0-1), social resources (scored 0-1) and basic social needs (scored 0-1); higher score of each domain means higher degree of frailty. Summed scores of 2 and >=3 from four domains (general resources, social behaviors, social resources and basic social needs) are then set as social pre-frailty and social frailty, respectively.

Models were adjusted for age, sex, study, alcohol consumption, smoking status and body mass index.

**Table S8.** Model selection for the multi-trajectories of physical, psychological and cognitive conditions.

| Number of groups | Polynomial degree | AIC | BIC | Probabilities of group membership (%) | Mean posterior probabilities | Posterior probabilities >0.7 (%) |
| --- | --- | --- | --- | --- | --- | --- |
| 2 | Physical conditions (1,1), Psychological symptoms (1,1), cognitive function (1,1) | -66191.59 | -66244.32 | 81.16/18/83 | 0.99/0.97 | 98.95/96.9 |
| 2 | Physical conditions (1,2), Psychological symptoms (1,2), cognitive function (1,2) | -66149.00 | -66211.61 | 81.31/18.69 | 0.99/0.97 | 99.09/96.45 |
| 2 | Physical conditions (2,2), Psychological symptoms (2,2), cognitive function (2,2) | -66114.63 | -66187.13 | 81.22/18.78 | 0.99/0.97 | 99.23/96.47 |
| 2 | Physical conditions (3,3), Psychological symptoms (3,3), cognitive function (3,3) | -66079.77 | -66172.04 | 81.46/18.54 | 0.99/0.97 | 99.09/96.42 |
| 3 | Physical conditions (1,1,1), Psychological symptoms (1,1,1), cognitive function (1,1,1) | -62107.55 | -62183.35 | 36.42/51.49/12.09 | 0.97/0.98/0.96 | 96.87/99.24/95.88 |
| 3 | Physical conditions (1,1,2), Psychological symptoms (1,2,2), cognitive function (1,2,2) | -63907.82 | -64000.09 | 31.54/51.28/17.18 | 0.90/0.93/0.96 | 86.73/92.19/95.27 |
| 3 | Physical conditions (2,2,2), Psychological symptoms (2,2,2), cognitive function (2,2,2) | -61770.17 | -61875.62 | 36.82/51.83/11.35 | 0.97/0.99/0.97 | 96.37/99.46/95.08 |
| 3 | Physical conditions (1,1,2), Psychological symptoms (2,3,3), cognitive function (2,2,3) | -61828.08 | -61936.83 | 36.95/51.62/11.43 | 0.97/0.99/0.97 | 96.78/99.32/95.28 |
| 4 | Physical conditions (1,1,1,1), Psychological symptoms (1,1,1,1), cognitive function (1,1,1,1) | -60947.62 | -61046.49 | 16.00/37.85/11.92/34.22 | 0.87/0.93/0.97/0.95 | 82.00/92.33/97.05/94.77 |
| **4** | **Physical conditions (1,1,2,2), Psychological symptoms (1,1,2,3), cognitive function (1,2,2,3)** | **-62638.44** | **-62766.96** | **27.22/47.50/16.18/9.10** | **0.88/0.94/0.96/0.88** | **83.26/90.64/94.33/84.43** |
| 4 | Physical conditions (1,2,2,2), Psychological symptoms (1,2,3,3), cognitive function (1,2,3,3) | -60738.42 | -60880.12 | 33.39/49.05/11.27/6.28 | 0.96/0.98/0.96/0.90 | 94.48/98.52/95.91/86.83 |
| 4 | Physical conditions (2,2,2,2), Psychological symptoms (2,2,3,3), cognitive function (2,2,3,3) | -62598.19 | -62749.78 | 27.31/47.41/16.18/9.10 | 0.88/0.92/0.96/0.88 | 83.85/90.45/94.88/83.33 |
| 4 | Physical conditions (3,3,3,3), Psychological symptoms (3,3,3,3), cognitive function (3,3,3,3) | -60607.55 | -60785.50 | 33.35/49.07/11.43/6.16 | 0.96/0.98/0.97/0.91 | 94.64/98.75/96.28/87.14 |
| 5 | Physical conditions (1,1,1,1,1), Psychological symptoms (1,1,1,1,1), cognitive function (1,1,1,1,1) | -60057.67 | -60179.60 | 15.76/30.51/36.13/11.58/6.02 | 0.86/0.94/0.93/0.96 | 80.61/92.23/91.56/95.87/85.16 |
| 5 | Physical conditions (1,1,1,2,2), Psychological symptoms (1,2,2,2,2), cognitive function (1,2,2,2,2) | -59754.36 | -59909.24 | 15.52/31.13/36.09/11.14/6.11 | 0.86/0.94/0.93/0.96/0.89 | 79.85/92.79/91.63/94.58/86.79 |
| 5 | Physical conditions (2,2,2,2,2), Psychological symptoms (2,2,2,2,2), cognitive function (2,2,2,2,2) | -59686.75 | -59858.11 | 15.83/30.78/36.30/11.07/6.02 | 0.87/0.94/0.93/0.96/0.89 | 82.61/92.31/91.54/94.55/85.62 |
| 5 | Physical conditions (2,2,3,3,3), Psychological symptoms (2,2,3,3,3), cognitive function (2,2,3,3,3) | -59603.42 | -59804.44 | 30.55/15.56/36.43/11.47/5.99 | 0.94/0.87/0.93/0.96/0.90 | 92.03/82.14/91.39/96.14/86.50 |
| 5 | Physical conditions (3,3,3,3,3), Psychological symptoms (3,3,3,3,3), cognitive function (3,3,3,3,3) | -59563.20 | -59783.99 | 15.76/31.10/35.93/11.29/5.92 | 0.86/0.94/0.93/0.96/0.90 | 81.89/92.59/91.21/95.45/88.16 |

AIC, Akaike information criterion; BIC, Bayesian information criteria.

**Table S9**. Baseline characteristics of included participants according to the trajectory of physical, psychological and cognitive conditions (n=5,382).

|  | Total (n=5382) | The trajectory of physical, psychological and cognitive conditions | | | | p-value |
| --- | --- | --- | --- | --- | --- | --- |
|  |  | Stably no physical, psychological or cognitive conditions (n=2585) | Increased physical conditions (n=882) | Increased physical and psychological conditions (n=469) | Increased physical and cognitive conditions  (n=1446) |  |
| **Age group (years)** |  |  |  |  |  | <0.001 |
| 45-54 | 1630 (30.3) | 951 (58.3) | 215 (13.2) | 161 (9.9) | 303 (18.6) |  |
| 55-64 | 2292 (42.6) | 1189 (51.9) | 355 (15.5) | 194 (8.5) | 554 (24.2) |  |
| 65-74 | 1175 (21.8) | 410 (34.9) | 250 (21.3) | 92 (7.8) | 423 (36.0) |  |
| 75- | 285 (5.3) | 35 (12.3) | 62 (21.8) | 22 (7.7) | 166 (58.2) |  |
| **Sex** |  |  |  |  |  | <0.001 |
| Man | 2688 (49.9) | 1187 (44.2) | 503 (18.7) | 144 (5.4) | 854 (31.8) |  |
| Woman | 2694 (50.1) | 1398 (51.9) | 379 (14.1) | 325 (12.1) | 592 (22.0) |  |
| **Current smoking** |  |  |  |  |  | <0.001 |
| No | 4294 (79.8) | 2119 (49.3) | 661 (15.4) | 386 (9.0) | 1128 (26.3) |  |
| Yes | 1088 (20.2) | 466 (42.8) | 221 (20.3) | 83 (7.6) | 318 (29.2) |  |
| **Alcohol consumption** |  |  |  |  |  | <0.001 |
| Less than weekly drinking | 2539 (47.2) | 1120 (44.1) | 454 (17.9) | 257 (10.1) | 708 (27.9) |  |
| Weekly drinking or more | 2843 (52.8) | 1465 (51.5) | 428 (15.1) | 212 (7.5) | 738 (26.0) |  |
| **Body mass index (kg/m2)** |  |  |  |  |  | <0.001 |
| <18.5 | 89 (1.7) | 39 (43.8) | 17 (19.1) | 9 (10.1) | 24 (27.0) |  |
| 18.5-23.9 | 2647 (49.2) | 1387 (52.4) | 352 (13.3) | 230 (8.7) | 678 (25.6) |  |
| 24.0-29.9 | 2036 (37.8) | 916 (45.0) | 374 (18.4) | 171 (8.4) | 575 (28.2) |  |
| 30.0- | 610 (11.3) | 243 (39.8) | 139 (22.8) | 59 (9.7) | 169 (27.7) |  |
| **Social frailty** |  |  |  |  |  | <0.001 |
| No | 3845 (71.4) | 2059 (53.6) | 575 (15.0) | 304 (7.9) | 907 (23.6) |  |
| Social pre-frailty | 1025 (19.0) | 370 (36.1) | 200 (19.5) | 104 (10.1) | 351 (34.2) |  |
| Social frailty | 512 (9.5) | 156 (30.5) | 107 (20.9) | 61 (11.9) | 188 (36.7) |  |
| **General resources** |  |  |  |  |  | <0.001 |
| No frailty | 1391 (25.8) | 448 (32.2) | 302 (21.7) | 137 (9.8) | 504 (36.2) |  |
| Frailty | 3991 (74.2) | 2137 (53.5) | 580 (14.5) | 332 (8.3) | 942 (23.6) |  |
| **Social behaviors** |  |  |  |  |  | <0.001 |
| No frailty | 2167 (40.3) | 865 (39.9) | 409 (18.9) | 208 (9.6) | 685 (31.6) |  |
| Frailty | 3215 (59.7) | 1720 (53.5) | 473 (14.7) | 261 (8.1) | 761 (23.7) |  |
| **Social resources** |  |  |  |  |  | 0.454 |
| No frailty | 683 (12.7) | 311 (45.5) | 119 (17.4) | 57 (8.3) | 196 (28.7) |  |
| Frailty | 4699 (87.3) | 2274 (48.4) | 763 (16.2) | 412 (8.8) | 1250 (26.6) |  |
| **Basic social needs** |  |  |  |  |  | <0.001 |
| No frailty | 1180 (21.9) | 463 (39.2) | 210 (17.8) | 156 (13.2) | 351 (29.7) |  |
| Frailty | 4202 (78.1) | 2122 (50.5) | 672 (16.0) | 313 (7.4) | 1095 (26.1) |  |

**Table S10**. The association between each measurement of social frailty domains and different trajectories of physical, psychological and cognitive conditions.

|  | Increased physical conditions (n=882) | Increased physical and psychological conditions (n=469) | Increased physical and cognitive conditions (n=1446) |
| --- | --- | --- | --- |
| **General resources** |  |  |  |
| Low educational level | 1.68 (1.38-2.04) | 1.36 (1.05-1.75) | 1.64 (1.38-1.95) |
| Low total household wealth | 3.21 (2.31-4.47) | 2.89 (1.94-4.30) | 5.30 (3.97-7.07) |
| **Social behaviors** |  |  |  |
| No monthly social activities | 1.52 (1.28-1.79) | 1.43 (1.16-1.76) | 1.87 (1.62-2.16) |
| Unemployment | 1.89 (1.01-3.56) | 2.05 (1.02-4.08) | 2.70 (1.59-4.57) |
| **Social resources** |  |  |  |
| Not married/partnered | 1.14 (0.89-1.47) | 0.85 (0.62-1.17) | 0.95 (0.76-1.18) |
| No living children | 0.88 (0.33-2.32) | 0.87 (0.30-2.50) | 1.14 (0.63-2.05) |
| **Basic social needs** |  |  |  |
| Not satisfied with life | 1.87 (1.50-2.32) | 3.19 (2.52-4.04) | 1.78 (1.48-2.14) |
| No weekly contact with children | 0.97 (0.69-1.38) | 0.69 (0.41-1.16) | 1.07 (0.80-1.43) |

OR, odds ratio; CI, confidence interval.

Models were adjusted for age, sex, study, alcohol consumption, smoking status and body mass index.

**Table S11**. The association between social frailty and different trajectories of physical, psychological and cognitive conditions after multiple imputation.

|  | Increased physical conditions (n=1153) | | Increased physical and psychological conditions (n=724) | | Increased physical and cognitive conditions (n=1866) | |
| --- | --- | --- | --- | --- | --- | --- |
|  | Events (%) | OR (95% CI) | Events (%) | OR (95% CI) | Events (%) | OR (95% CI) |
| **Social frailty** |  |  |  |  |  |  |
| No | 711 (14.4) | Ref | 445 (9.0) | Ref | 1155 (23.4) | Ref |
| Social pre-frailty | 273 (19.4) | 1.75 (1.47-2.09) | 175 (12.5) | 1.88 (1.53-2.30) | 435 (31.0) | 1.75 (1.50-2.04) |
| Social frailty | 169 (21.7) | 2.46 (1.96-3.10) | 104 (13.4) | 2.41 (1.86-3.13) | 276 (35.5) | 2.50 (2.05-3.06) |
| Per score increase | / | 1.44 (1.34-1.54) | / | 1.42 (1.31-1.54) | / | 1.45 (1.37-1.54) |
| **Domains of social frailty** |  |  |  |  |  |  |
| General resources | 423 (21.3) | 2.04 (1.74-2.38) | 220 (11.1) | 1.64 (1.37-1.98) | 689 (34.7) | 2.15 (1.88-2.46) |
| Social behaviors | 590 (19.0) | 1.66 (1.44-1.92) | 346 (11.2) | 1.52 (1.28-1.80) | 938 (30.3) | 1.76 (1.56-2.00) |
| Social resources | 155 (17.8) | 1.13 (0.91-1.40) | 95 (10.9) | 1.02 (0.79-1.30) | 240 (27.5) | 0.96 (0.79-1.15) |
| Basic social needs | 301 (18.8) | 1.67 (1.41-1.97) | 237 (14.8) | 2.21 (1.84-2.66) | 435 (27.2) | 1.34 (1.16-1.55) |

OR, odds ratio; CI, confidence interval.

Social frailty is measured by four domains: general resources (scored 0-1), social behaviors (scored 0-1), social resources (scored 0-1) and basic social needs (scored 0-1); higher score of each domain means higher degree of frailty. Summed scores of 2 and >=3 from four domains (general resources, social behaviors, social resources and basic social needs) are then set as social pre-frailty and social frailty, respectively.

Models were adjusted for age, sex, study, alcohol consumption, smoking status and body mass index.

**Table S12**. The association between social frailty and different trajectories of physical, psychological and cognitive conditions by age.

|  | OR (95% CI) for physical, psychological and cognitive trajectories | | |
| --- | --- | --- | --- |
|  | Increased physical conditions (n=882) | Increased physical and psychological conditions (n=469) | Increased physical and cognitive conditions  (n=1446) |
| ***<65 years*** |  |  |  |
| **Social frailty** |  |  |  |
| No | Ref | Ref | Ref |
| Social pre-frailty | 1.79 (1.39, 2.30) | 2.13 (1.60, 2.83) | 2.32 (1.89, 2.85) |
| Social frailty | 2.61 (1.87, 3.64) | 2.66 (1.84, 3.85) | 2.72 (2.05, 3.62) |
| Per score increase | 1.46 (1.32, 1.6) | 1.55 (1.39, 1.73) | 1.60 (1.47, 1.74) |
| **Domains of social frailty** |  |  |  |
| General resources | 2.21 (1.78, 2.75) | 1.95 (1.50, 2.52) | 2.40 (2.00, 2.89) |
| Social behaviors | 1.55 (1.28, 1.88) | 1.60 (1.27, 2.02) | 2.01 (1.70, 2.37) |
| Social resources | 1.18 (0.85, 1.63) | 1.03 (0.72, 1.48) | 0.89 (0.67, 1.17) |
| Basic social needs | 1.61 (1.27, 2.04) | 2.64 (2.05, 3.41) | 1.72 (1.41, 2.09) |
| ***>=65 years*** |  |  |  |
| **Social frailty** |  |  |  |
| No | Ref | Ref | Ref |
| Social pre-frailty | 1.83 (1.26, 2.66) | 0.94 (0.53, 1.64) | 1.71 (1.23, 2.38) |
| Social frailty | 2.30 (1.33, 3.97) | 1.51 (0.71, 3.20) | 3.01 (1.85, 4.88) |
| Per score increase | 1.43 (1.23, 1.66) | 1.08 (0.87, 1.34) | 1.48 (1.30, 1.69) |
| **Domains of social frailty** |  |  |  |
| General resources | 2.34 (1.65, 3.32) | 1.36 (0.82, 2.26) | 2.61 (1.91, 3.55) |
| Social behaviors | 1.53 (1.10, 2.12) | 0.96 (0.60, 1.54) | 1.62 (1.22, 2.16) |
| Social resources | 1.34 (0.91, 1.98) | 0.62 (0.34, 1.13) | 1.33 (0.95, 1.85) |
| Basic social needs | 1.34 (0.94, 1.91) | 1.53 (0.95, 2.47) | 0.98 (0.72, 1.34) |

OR, odds ratio; CI, confidence interval.

Social frailty is measured by four domains: general resources (scored 0-1), social behaviors (scored 0-1), social resources (scored 0-1) and basic social needs (scored 0-1); higher score of each domain means higher degree of frailty. Summed scores of 2 and >=3 from four domains (general resources, social behaviors, social resources and basic social needs) are then set as social pre-frailty and social frailty, respectively.

Models were adjusted for age, sex, study, alcohol consumption, smoking status and body mass index.

**Table S13**. The association between social frailty and different trajectories of physical, psychological and cognitive conditions by sex.

|  | OR (95% CI) for physical, psychological and cognitive trajectories | | |
| --- | --- | --- | --- |
|  | Increased physical conditions (n=882) | Increased physical and psychological conditions (n=469) | Increased physical and cognitive conditions  (n=1446) |
| ***Male*** |  |  |  |
| **Social frailty** |  |  |  |
| No | Ref | Ref | Ref |
| Social pre-frailty | 1.87 (1.42, 2.47) | 1.85 (1.20, 2.87) | 1.89 (1.48, 2.41) |
| Social frailty | 2.20 (1.44, 3.36) | 2.57 (1.41, 4.68) | 2.62 (1.82, 3.78) |
| Per score increase | 1.49 (1.33, 1.67) | 1.55 (1.31, 1.84) | 1.52 (1.38, 1.68) |
| **Domains of social frailty** |  |  |  |
| General resources | 2.40 (1.86, 3.09) | 1.93 (1.29, 2.89) | 2.23 (1.78, 2.79) |
| Social behaviors | 1.55 (1.24, 1.94) | 1.81 (1.26, 2.60) | 1.87 (1.54, 2.28) |
| Social resources | 1.10 (0.75, 1.61) | 0.73 (0.37, 1.44) | 1.05 (0.76, 1.45) |
| Basic social needs | 1.54 (1.18, 2.01) | 2.21 (1.51, 3.25) | 1.36 (1.08, 1.71) |
| ***Female*** |  |  |  |
| **Social frailty** |  |  |  |
| No | Ref | Ref | Ref |
| Social pre-frailty | 1.67 (1.23, 2.27) | 1.70 (1.24, 2.33) | 2.27 (1.76, 2.94) |
| Social frailty | 2.38 (1.63, 3.48) | 2.20 (1.48, 3.28) | 2.70 (1.93, 3.77) |
| Per score increase | 1.35 (1.20, 1.52) | 1.36 (1.21, 1.53) | 1.55 (1.40, 1.71) |
| **Domains of social frailty** |  |  |  |
| General resources | 1.77 (1.36, 2.32) | 1.63 (1.23, 2.16) | 2.37 (1.88, 2.98) |
| Social behaviors | 1.57 (1.22, 2.01) | 1.30 (1.01, 1.68) | 1.95 (1.57, 2.41) |
| Social resources | 1.17 (0.85, 1.62) | 0.89 (0.62, 1.27) | 0.92 (0.69, 1.22) |
| Basic social needs | 1.64 (1.22, 2.20) | 2.52 (1.90, 3.33) | 1.65 (1.29, 2.12) |

OR, odds ratio; CI, confidence interval.

Social frailty is measured by four domains: general resources (scored 0-1), social behaviors (scored 0-1), social resources (scored 0-1) and basic social needs (scored 0-1); higher score of each domain means higher degree of frailty. Summed scores of 2 and >=3 from four domains (general resources, social behaviors, social resources and basic social needs) are then set as social pre-frailty and social frailty, respectively.

Models were adjusted for age, sex, study, alcohol consumption, smoking status and body mass index.

**Table S14**. Meta-analysis for the association between social frailty and different trajectories of physical, psychological and cognitive conditions by study.

|  | Increased physical conditions (n=882) | Increased physical and psychological conditions (n=469) | Increased physical and cognitive conditions  (n=1446) |
| --- | --- | --- | --- |
| ***HRS*** |  |  |  |
| No | Ref | Ref | Ref |
| Social pre-frailty | 2.13 (0.47, 9.74) | 1.02 (0.09, 11.90) | 4.28 (1.37, 13.35) |
| Social frailty | 3.74 (0.59, 23.60) | 4.82 (0.36, 65.09) | 3.71 (0.80, 17.18) |
| Per score increase | 1.59 (0.92, 2.76) | 1.47 (0.63, 3.45) | 1.97 (1.27, 3.05) |
| ***ELSA*** |  |  |  |
| No | Ref | Ref | Ref |
| Social pre-frailty | 2.17 (1.31, 3.61) | 2.06 (1.20, 3.55) | 1.71 (1.18, 2.48) |
| Social frailty | 2.61 (1.26, 5.43) | 3.01 (1.49, 6.08) | 2.47 (1.48, 4.12) |
| Per score increase | 1.43 (1.17, 1.75) | 1.60 (1.30, 1.96) | 1.38 (1.20, 1.59) |
| ***SHARE*** |  |  |  |
| No | Ref | Ref | Ref |
| Social pre-frailty | 1.79 (1.36, 2.37) | 1.61 (1.15, 2.25) | 1.96 (1.53, 2.53) |
| Social frailty | 2.45 (1.72, 3.49) | 2.07 (1.36, 3.14) | 2.63 (1.90, 3.65) |
| Per score increase | 1.45 (1.30, 1.62) | 1.36 (1.20, 1.54) | 1.52 (1.38, 1.68) |
| ***CHARLS*** |  |  |  |
| No | Ref | Ref | Ref |
| Social pre-frailty | 1.71 (1.14, 2.58) | 1.88 (1.05, 3.38) | 2.51 (1.75, 3.60) |
| Social frailty | 1.95 (0.96, 3.97) | 2.09 (0.79, 5.53) | 2.85 (1.53, 5.32) |
| Per score increase | 1.41 (1.19, 1.68) | 1.43 (1.11, 1.84) | 1.71 (1.46, 2.01) |
| ***Pooled*** |  |  |  |
| Social pre-frailty |  |  |  |
| OR (95%CI) | 1.83 (1.49, 2.25) | 1.74 (1.35, 2.25) | 2.08 (1.68, 2.58) |
| Heterogeneity (*I*^2^) | 0% | 0% | 22% |
| Social frailty |  |  |  |
| OR (95%CI) | 2.41 (1.81, 3.21) | 2.29 (1.64, 3.20) | 2.65 (2.07, 3.40) |
| Heterogeneity (*I*^2^) | 0% | 0% | 0% |
| Per score increase |  |  |  |
| OR (95%CI) | 1.44 (1.33, 1.57) | 1.42 (1.29, 1.57) | 1.54 (1.39, 1.71) |
| Heterogeneity (*I*^2^) | 0% | 0% | 43% |

OR, odds ratio; CI, confidence interval; HRS, US Health and Retirement Study; ELSA, English Longitudinal Study on Ageing; SHARE, Survey of Health, Ageing and Retirement in Europe; CHARLS, China Health and Retirement Longitudinal Study.

Social frailty is measured by four domains: general resources (scored 0-1), social behaviors (scored 0-1), social resources (scored 0-1) and basic social needs (scored 0-1); higher score of each domain means higher degree of frailty. Summed scores of 2 and >=3 from four domains (general resources, social behaviors, social resources and basic social needs) are then set as social pre-frailty and social frailty, respectively.

Models were adjusted for age, sex, study, alcohol consumption, smoking status and body mass index.


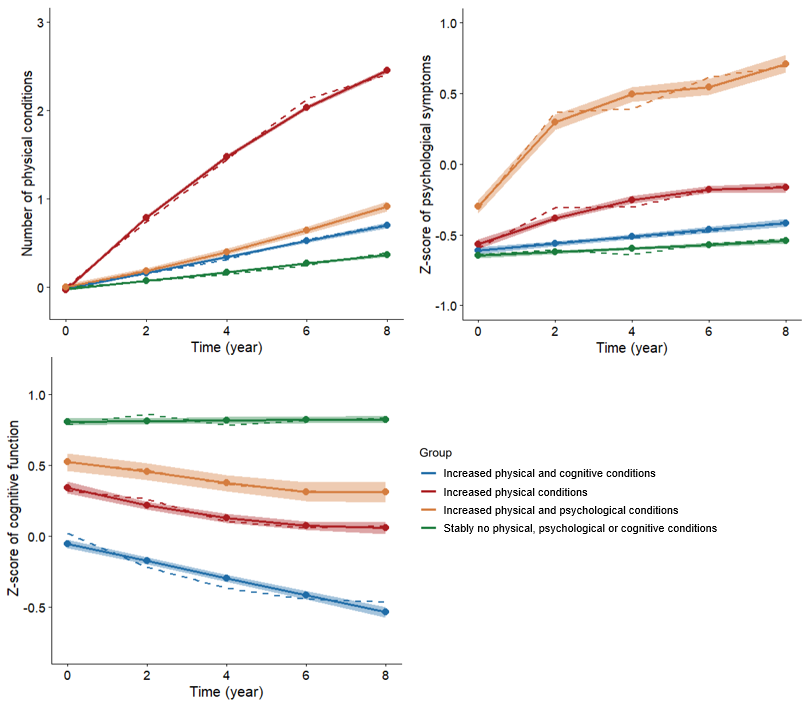
 **Figure S1**. Multi-trajectories of physical, psychological and cognitive conditions after multiple imputation (N=7,119).
